# Prediction of Peptide and Protein Propensity for Amyloid Formation

**DOI:** 10.1371/journal.pone.0134679

**Published:** 2015-08-04

**Authors:** Carlos Família, Sarah R. Dennison, Alexandre Quintas, David A. Phoenix

**Affiliations:** 1 School of Forensic and Investigative Science, University of Central Lancashire, Preston, PR1 2HE, United Kingdom; 2 Centro de Investigação Interdisciplinar Egas Moniz, Instituto Superior de Ciências da Saúde Egas Moniz, Campus Universitário, Quinta, Da Granja, Monte de Caparica, 2829–511, Caparica, Portugal; 3 Research and Innovation Office, UCLan Biomedical Research Facility, University of Central Lancashire, Preston, PR1 2HE, United Kingdom; 4 School of Applied Science, London South Bank University, 103 Borough Road, London, SE1 0AA, United Kingdom; Russian Academy of Sciences, Institute for Biological Instrumentation, RUSSIAN FEDERATION

## Abstract

Understanding which peptides and proteins have the potential to undergo amyloid formation and what driving forces are responsible for amyloid-like fiber formation and stabilization remains limited. This is mainly because proteins that can undergo structural changes, which lead to amyloid formation, are quite diverse and share no obvious sequence or structural homology, despite the structural similarity found in the fibrils. To address these issues, a novel approach based on recursive feature selection and feed-forward neural networks was undertaken to identify key features highly correlated with the self-assembly problem. This approach allowed the identification of seven physicochemical and biochemical properties of the amino acids highly associated with the self-assembly of peptides and proteins into amyloid-like fibrils (normalized frequency of β-sheet, normalized frequency of β-sheet from LG, weights for β-sheet at the window position of 1, isoelectric point, atom-based hydrophobic moment, helix termination parameter at position j+1 and ΔG° values for peptides extrapolated in 0 M urea). Moreover, these features enabled the development of a new predictor (available at http://cran.r-project.org/web/packages/appnn/index.html) capable of accurately and reliably predicting the amyloidogenic propensity from the polypeptide sequence alone with a prediction accuracy of 84.9 % against an external validation dataset of sequences with experimental *in vitro*, evidence of amyloid formation.

## Introduction

Amyloid fiber formation has long been associated with several debilitating diseases and in 2014 there were approximately fifty reported human diseases linked to amyloid [1]. These include localized amyloidosis such as pancreatic amyloidosis, atrial amyloidosis of the heart, Alzheimer’s disease, Parkinson’s disease, Huntington’s disease and Creutzfeldt-Jakob’s disease [2,3], as well as systemic diseases such as familial amyloid polyneuropathy or immunoglobulin light-chain amyloidosis [3,4]. Amyloid diseases normally arise due to failures in the dedicated aggregation prevention quality control systems, which exist *in vivo*. Such systems range from simple protein sequence determinants that evolved over generations, to more complex cellular machinery, such as that involved in heat-shock response, unfolded-protein response, endoplasmic-reticulum associated degradation and autophagy, among others [5].

Over the last two decades several publications have shown that amyloid could be produced through “controlled” fibrillization and with specific biological functions instead of an off-pathway product of protein folding that leads to disease [6]. Examples include the bacterial pili [7], curly fibrils expressed in *Escherichia coli* and *Salmonella* (involved in surface colonization and biofilm formation) [8], human pigment binding templates [9] and regulation of the expression reading-through stop-codon in yeast (*Saccharomyces cerevisiae*, Sup35p) [6]. Amyloid formation is also involved in providing a storage mechanism for several hormones in secretory granules [10] and structural and protective functions in the eggshell of many fish and insects [11–13].

Protein aggregation and subsequent assembly into amyloid like structures is commonly seen as a major problem in large scale expression of peptides and proteins of potential interest within the field of biotechnology. Usually these proteins are recombinant polypeptides from mammalian and viral heterologous genes, which tend to adopt irregular or incomplete folds when overexpressed in prokaryotic hosts. This frequently results in the accumulation of the protein as insoluble aggregates within inclusion bodies, reducing the yield of extraction and purification, and ultimately, the economic viability of the purification process [14]. In contrast, recent work has exploited protein aggregation into amyloid fibrils and subsequent accumulation in inclusion bodies with the purpose of improving protein expression. This is based on the assumption that protein storage in inclusion bodies reduces the protein concentration in the cytoplasm, isolating the proteins from the cytoplasmic content and thus protecting them against proteolysis and other degradation pathways [15].

In the area of material science, amyloid fibrils are seen as an important source of innovation, since they may provide insights into a wide range of properties that could be explored in the design of new nanomaterials. The ability of amyloid to self-assemble or self-replicate into well-defined structures, their nanoscale dimensions, the diversity of associated protein sequences, the ease of production and low cost make them key systems for investigation [5,13,16]. Indeed amyloid fiber formation has already been used as a “bottom-up” approach for the fabrication of a wide range of nanostructured materials, from isolated fibers for the construction of synthetic monomolecular wires [12] and biotemplated metal wires to be used in nanoscale electrical circuitry [17], to ordered amyloid monolayers for the construction of templates for mineralization and directed crystal growth, or scaffolds for drug delivery and tissue engineering applications [12,18].

Amyloid fibers are unbranched filamentous protein aggregates with an indefinite length and a diameter that can range from 6 to 12 nm [19]. They are commonly formed by polypeptide chains arranged in a characteristic cross-β conformation with strands perpendicularly oriented to the fiber long axis. This structure results in a series of stacked β-chains that propagate along the fiber [19–21]. Polypeptides within the amyloid fibers are thus arranged in a highly ordered fashion [22]. Despite this structural similarity [23], proteins that can undergo structural changes that ultimately lead to amyloid formation are quite diverse, sharing no obvious sequential or structural homology [24]. Furthermore, a number of researchers have suggested that the ability to form amyloid fibrils is an intrinsic property of the polypeptide backbone [7,24–28]. In fact, it has been shown that many proteins under the appropriate environmental conditions (concentration, ionic strength, temperature, etc.), can aggregate into highly ordered fibrillar structures [7,24,28] forming the tight packed steric zipper that constitutes the core of the protofilament [29]. Under physiological conditions, however, even at high concentrations, the majority of soluble proteins will remain in solution, while hydrophobic proteins usually tend to form amorphous aggregates [29] and only a relatively small number of proteins actually undergo amyloid fiber formation [7]. This degree of specificity points towards protein sequence details and native-state integrity or structural stability as major determinants of how easily proteins are able to adopt an amyloid structure under specific environmental conditions [30–32].

Due to the relevance of amyloid in such diverse areas of study as biochemistry, medicine, microbiology, biotechnology and materials science, the knowledge of which and how peptides and proteins undergo amyloid formation is of paramount importance. Experimental identification of amyloidogenic proteins *in vitro* is extremely laborious and time-consuming. Hence, computational approaches that can accurately and reliably predict the amyloidogenic propensity of peptides and assess their amyloidogenic potential based on the sequence information alone are extremely valuable. Additionally, such work can help to elucidate key driving forces responsible for amyloid-like fiber formation and stabilization and provide new insights into the self-assembly problem.

Over the last decade, several computational algorithms have been developed [29,33–38]. These follow two major approaches in order to predict the aggregation propensity of proteins into amyloid fibrils and to identify within the sequence, regions more prone to form fibrils [5,39]. Computational algorithms can thus be classified as: i) empirical or sequence based methods that rely on physicochemical and biochemical properties of the amino acids [39], or ii) structure-based methods that normally combine the sequence based methods with three dimensional structural information gathered from atomistic simulation of the protein segments with the crystallographic structure of short fibril forming peptides [39].

Herein, we report the development of a new phenomenological amyloidogenicity propensity predictor based on a machine learning approach through recursive feature selection and feed-forward neural networks, taking advantage of all newly published sequences with experimental, *in vitro*, evidence of amyloid formation. This approach relies on the assumptions that: i) small peptide stretches within an amyloidogenic protein can act as amyloid forming facilitators that will eventually direct the refolding of the protein along a path involving the formation of an energetically favourable amyloid conformation [40,41] and ii) the minimum length of these facilitator sequences or hot spots comprises six amino acids, given that in the literature there are a large number of hexapeptides with *in vitro* experimental evidence of amyloid formation. These are reflected in the *in silico* experimental procedure undertaken, where recursive feature selection and neural network training was performed through a dataset of six amino acid sequences while the external validation of the trained neural network was performed with a dataset of peptides and proteins with lengths greater than six amino acids, although using a sliding window of six amino acids.

Recursive feature selection plays a fundamental role, by relieving the artificial neural networks learning algorithm from the “curse of dimensionality” [42]. This diminishes the number of features composing the input vectors and thus improves its ability to learn. For our selected predictor feature selection identified seven key physicochemical and biochemical features of the amino acids, which are highly related to the self-assembly of peptides and proteins into amyloid fibers. These included the normalized frequency of β-sheet [43], normalized frequency of β-sheet from a dataset of 44 sample proteins named LG [44], first order neural network neuronal weights for β-sheet at position 1 of a 13 amino acids length window [45], isoelectric point [46], atom-based hydrophobic moment [47], helix termination parameter or theoretical estimate of helix-coil stability parameter for the natural occurring amino acids when found at position j+1 of the C-terminal region of the helix [48] and ΔG° values for peptides extrapolated to 0 M urea from a two-state model derived from urea denaturation curves that correlate the dissociation constants of peptides containing one of 20 natural occurring amino acids in a guest position, with the urea concentration [49]. All of these factors relate to biophysical properties which have been consistently pointed out in the literature as fundamental factors in the molecular mechanism of amyloid formation and are proved here to have a high correlation with the ability of a sequence to undergo amyloid formation [7,37,50–53].

The developed predictor based on these physicochemical and biochemical characteristics of the amino acids proved able to accurately and reliably predict amyloidogenic propensity from the polypeptide sequence alone and identify hot spots within these sequences. Comparison with other published amyloidogenic propensity prediction methods (Aggrescan [34], AMYLPRED [37], AMYLPRED2 [54], FoldAmyloid [38], MetAmyl [55], Pafig [39],Pasta [35], Pasta2 [56], Tango [33], Waltz [29] and Zyggregator [36]) showed a high accuracy value based on the classification of the training dataset (78.0%) and was only outperformed by MetAmyl (79.1%). However, it obtained the highest accuracy value based on the classification of an external validation sequence dataset (84.9%), outperforming all methods, including MetAmyl (83.4%).

## Results

### Orthogonal vectors based artificial neural networks

In order to establish an internal reference predictor based on amino acids present within the sequences, and their relative order, several artificial neural networks were trained based on input vectors computed through an orthogonal encoding of the amino acids present. The neural network with the highest accuracy was selected and showed an overall prediction accuracy of 82.8% in the classification of the training sequence dataset, with a sensitivity of 83.0%, a specificity of 82.6%, a positive predictive value of 80.0% and a negative predictive value of 85.3%. Classification of the external validation sequences dataset rendered an overall accuracy of 73.1%, a sensitivity of 90.6%, and a specificity of 31.0%, with a positive predictive value of 75.9%, and a negative predictive value of 57.9%.

### Physicochemical and biochemical based artificial neural networks

A physicochemical and biochemical description of the polypeptide sequences was created through encoding the sequence based on the APDBase [57] and AAindex [58,59] databases of physicochemical and biochemical properties of the amino acids. The computed features vectors were then submitted to recursive feature selection. This resulted in 96, 10, 13, 548 and 810 selected features for the input vectors computed through the APDBase encoding, and 109, 14, 334, 100 and 969 selected features for the input vectors computed through the AAindex encoding, for the internal classifiers of random forests (rf), naïve bayes (nb), support vector machines (svm), shrinkage discriminant analysis (sda) and sparse partial least squares (spls), respectively.

Artificial neural networks were trained using newly computed input vectors based on these selected features. For neural networks trained with feature vectors computed through APDBase encoding, the values obtained for the overall accuracy range between 76.7% (NN_APD_sda) to 86.8% (NN_APD_svm), for sensitivity from 73.5% (NN_APD_sda) to 87.9% (NN_APD_svm) and specificity from 77.7% (NN_APD_nb) to 86.5% (NN_APD_svm) (Table 1). For neural networks trained with feature vectors computed through AAindex encoding, the overall accuracy values obtained range from 78.0% (NN_AA_rf) to 91.9% (NN_AA_sda), sensitivity from 80.1% (NN_AA_svm) to 87.4% (NN_AA_nb) and specificity from 70.2% (NN_AA_nb) to 95.6% (NN_AA_sda) (Table 1).

**Table 1:** Training sequences dataset classification results (%) for the selected neural networks obtained through APDBase or AAindex encoding, after feature selection with one of the internal classifiers, rf, nb, svm, sda and spls. Where SI is the sensitivity, SP the specificity, PPV the positive predictive value, NPV the negative predictive value and AC the overall accuracy, averaged after 10-fold stratified resampling.

		SI	SP	PPV	NPV	AC
**APDBase**	**NN_APD_rf**	82.6	85.0	82.3	85.1	83.8
	**NN_APD_nb**	77.1	77.7	74.1	80.3	77.4
	**NN_APD_svm**	87.9	86.5	84.3	89.0	86.8
	**NN_APD_sda**	73.5	80.0	75.4	78.9	76.7
	**NN_APD_spls**	80.4	79.1	76.5	82.4	79.7
**AAindex**	**NN_AA_rf**	84.0	86.4	83.9	86.4	85.1
	**NN_AA_nb**	87.4	70.2	71.2	86.9	78.0
	**NN_AA_svm**	80.1	76.9	75.1	82.8	78.7
	**NN_AA_sda**	86.8	95.6	94.9	90.3	91.9
	**NN_AA_spls**	81.0	83.3	80.1	84.2	81.7

Classification of the external validation sequences dataset for neural networks trained with the selected features vectors computed through the APDBase encoding, showed high overall accuracy for 4 of the 5 neural networks selected. The most effective neural network had an accuracy of 83.0% (NN_APD_rf), a sensitivity of 89.4% and a specificity of 67.8%. While for neural networks trained with the selected features vectors computed through the AAindex encoding, only 2 of the 5 neural networks selected showed high overall accuracy. The best accuracy shown was 84.9% (NN_AA_nb) with a sensitivity of 87.4% and a specificity of 78.9%, and the second best was 82.2% (NN_AA_rf), with a sensitivity of 90.8% and a specificity of 62.6% (Table 2).

**Table 2:** External validation sequences dataset classification results (%) for the selected neural networks obtained through APDBase and AAindex encoding, after feature selection with one of the internal classifiers, rf, nb, svm, sda and spls. Where SI is the sensitivity, SP the specificity, PPV the positive predictive value, NPV the negative predictive value and AC the overall accuracy, averaged after 10-fold stratified resampling.

		SI	SP	PPV	NPV	AC
**APDBase**	**NN_APD_rf**	89.4	67.8	86.8	73.5	83.0
	**NN_APD_nb**	78.7	82.5	91.3	62.4	79.9
	**NN_APD_svm**	91.8	40.1	78.7	68.2	76.6
	**NN_APD_sda**	91.9	16.3	72.7	46.2	69.8
	**NN_APD_spls**	94.1	17.9	73.1	57.6	71.4
**AAindex**	**NN_AA_rf**	90.8	62.6	85.3	73.4	82.2
	**NN_AA_nb**	87.4	78.9	90.8	72.3	84.9
	**NN_AA_svm**	95.6	12.6	72.4	51.0	71.2
	**NN_AA_sda**	93.5	15.7	72.6	52.1	70.4
	**NN_AA_spls**	93.9	12.5	72.1	48.9	70.0

### Analysis of the amyloidogenicity propensity prediction selected artificial neural network

The artificial neural network based on the description of the polypeptide sequences through the physicochemical and biochemical properties of the amino acids that showed the highest overall accuracy in the classification of the external validation sequences dataset (NN_AA_nb) was selected for further analysis, and will hereafter be referred as APPNN, standing for Amyloidogenicity Propensity Prediction Neural Network. This neural network was trained with a small subset of 14 features selected from the input vectors computed through the AAindex database of physicochemical and biochemical properties of the amino acids, by the Naïve Bayes classifier embedded within the recursive feature selection algorithm. Identification of these features revealed that three consisted of summation properties of the amino acid of the Normalized frequency of β-sheet [43], the Normalized frequency of β-sheet from LG [44] and the Weights for β-sheet at the window position of 1 [45]. Another two consisted of the values of standard deviation and range of the Isoelectric Point [46], while a further nine consisted of the standard deviation, range and mean absolute deviation of the Atom-based hydrophobic moment [47], the Helix termination parameter at position j+1 [48] and the ΔG° values for the peptides extrapolated to 0 M urea [49].

A comparative analysis between the APPNN and several published prediction methods (Aggrescan, AMYLPRED, AMYLPRED2, FoldAmyloid, MetAmyl, Pafig, Pasta, Pasta2, Tango, Waltz and Zyggregator), was undertaken for the classification of both training and external validation sequences datasets to assess if differences existed and if so if they were statistically significant. Prediction results were used, after bootstrapping, to compute the values of sensitivity, specificity, positive predictive value, negative predictive value and accuracy, with corresponding 95% confidence intervals; and after 10-fold stratified resampling, to determine if the accuracy values obtained for all predictors were sampled from populations with identical distributions (case in which all differences between groups are due to random sampling) through Friedman’s test.

The results obtained for the classification of the training sequences dataset (Table 3), shown for APPNN, high values of specificity (70.2%) and positive prediction value (71.1%), although these were surpassed by several other methods. In contrast, APPNN showed the highest values for sensitivity (87.4%) and negative predictive value (86.9%). APPNN also shown a high accuracy value (78.0%), only outperformed by the Metamyl predictor (79.1%). Friedman’s test showed differences between predictors’ accuracy to be statistically significant (chi-squared = 62.7812, df = 11, p = 2.81E-09) with pairwise comparisons identifying key differences between the APPNN and the methods Aggrescan, Foldamyloid and Tango and Zyggregator (p < 0.05).

**Table 3:** Results obtained for the classification of the training sequences dataset for each predictor, where TP corresponds to the number of true positives, TN to the number of true negatives, FP to the number of false positives and FN to the number of false negatives. The values of sensitivity, specificity, positive predictive value (PPV), negative predictive value (NPV) and accuracy, with corresponding 95% confidence intervals, were obtained using bootstrap replicates. The p-value corresponds to the p-value obtained for the comparison of the accuracy values between the APPNN and each given other predictors using the Wilcoxon-Nemenyi-McDonald-Thompson post-hoc test performed after 10-fold stratified resampling.

	TP	TN	FP	FN	Sensitivity [95% CI]	Specificity [95% CI]	PPV [95% CI]	NPV [95% CI]	Accuracy [95% CI]	p-value
**APPNN**	118	113	48	17	87.4 [80.6, 92.0]	70.2 [62.7, 77.0]	71.1 [63.7, 77.7]	86.9 [79.9, 91.9]	78.0 [72.6, 82.4]	-
**Aggrescan**	77	111	50	58	57.0 [48.5, 65.0]	68.9 [60.8, 75.6]	60.6 [51.6, 68.4]	65.7 [58.1, 73.0]	63.5 [57.1, 68.2]	0.03
**Amylpred**	68	141	20	67	50.4 [42.0, 58.7]	87.6 [81.4, 91.8]	77.3 [67.9, 84.7]	67.8 [61.4, 74.6]	70.6 [64.5, 75.3]	0.52
**Amylpred2**	77	141	20	58	57.0 [48.3, 64.7]	87.6 [81.2, 91.9]	79.4 [70.2, 86.3]	70.9 [63.9, 77.0]	73.6 [67.6, 78.0]	1.00
**Foldamyloid**	114	55	106	21	84.4 [77.7, 89.8]	34.2 [26.9, 42.1]	51.8 [44.6, 58.3]	72.4 [61.3, 81.8]	57.1 [50.7, 62.4]	1.48E-04
**Metamyl**	104	130	31	31	77.0 [69.7, 83.6]	80.7 [74.0, 86.3]	77.0 [69.4, 83.6]	80.7 [74.5, 86.4]	79.1 [74.0, 83.1]	1.00
**Pafig**	115	94	67	20	85.2 [78.5, 90.4]	58.4 [50.4, 65.7]	63.2 [55.8, 69.6]	82.5 [74.8, 88.4]	70.6 [64.7, 75.0]	0.49
**Pasta**	84	130	31	51	62.2 [53.8, 70.2]	80.7 [73.8, 86.6]	73.0 [63.4, 80.2]	71.8 [64.9, 78.0]	72.3 [66.2, 76.7]	0.94
**Pasta2**	82	127	34	53	60.7 [52.1, 68.6]	78.9 [71.9, 84.6]	70.7 [61.0, 78.0]	70.6 [63.5, 76.8]	70.6 [64.3, 75.0]	0.54
**Tango**	6	158	3	129	4.4 [1.5, 9.1]	98.1 [94.8, 99.4]	66.7 [20.0, 92.9]	55.1 [49.3, 60.9]	55.4 [49.3, 60.8]	3.08E-05
**Waltz**	91	129	32	44	67.4 [59.6, 75.0]	80.1 [73.4, 85.5]	74.0 [65.9, 81.1]	74.6 [67.8, 81.3]	74.3 [68.9, 78.7]	0.96
**Zyggregator**	100	91	70	35	74.1 [65.6, 79.8]	56.5 [48.4, 63.6]	58.8 [51.3, 65.8]	72.2 [63.6, 79.4]	64.5 [58.4, 69.3]	0.01

The results obtained for classification of the external validation sequences dataset (Table 4), shown APPNN had high values of sensitivity (87.4%), specificity (78.9%), positive predictive value (90.9%) and negative predictive value (72.3%). However it was only for the accuracy value (84.9%) that APPNN was able to outperform all other prediction methods. Friedman’s test again showed that the differences between predictors’ accuracy was statistically significant (chi-squared = 78.1777, df = 11, p-value = 3.318E-12) with pairwise comparison confirming superior performance of APPNN compared to Pasta, Pasta2, Tango, Waltz and Zyggregator (p < 0.05).

**Table 4:** Results obtained for the classification of the external validation sequence dataset for each predictor, where TP corresponds to the number of true positives, TN to the number of true negatives, FP to the number of false positives and FN to the number of false negatives. The values of sensitivity, specificity, positive predictive value (PPV), negative predictive value (NPV) and accuracy, with corresponding 95% confidence intervals, were obtained using bootstrap replicates. The p-value corresponds to the p-value obtained for the comparison of the accuracy values between the APPNN and each given predictor using the Wilcoxon-Nemenyi-McDonald-Thompson post-hoc test performed after 10-fold stratified resampling.

	TP	TN	FP	FN	Sensitivity [95% CI]	Specificity [95% CI]	PPV [95% CI]	NPV [95% CI]	Accuracy [95% CI]	p-value
**APPNN**	298	112	30	43	87.4 [83.4, 90.6]	78.9 [70.9, 85.0]	90.9 [87.2, 93.6]	72.3 [64.9, 78.8]	84.9 [81.2, 87.6]	-
**Aggrescan**	284	97	45	57	83.3 [78.9, 87.0]	68.3 [59.7, 75.7]	86.3 [82.1, 89.7]	63.0 [55.1, 70.6]	78.9 [74.9, 82.4]	0.90
**Amylpred**	248	120	22	93	72.7 [68.1, 77.3]	84.5 [77.9, 89.9]	91.9 [88.2, 94.7]	56.3 [49.3, 63.0]	76.2 [72.3, 79.7]	0.51
**Amylpred2**	271	122	20	70	79.5 [75.3, 83.7]	85.9 [79.6, 90.9]	93.1 [89.9, 95.7]	63.5 [56.4, 70.3]	81.4 [77.6, 84.7]	1.00
**Foldamyloid**	306	74	68	35	89.7 [86.3, 92.5]	52.1 [43.9, 60.0]	81.8 [77.6, 85.5]	67.9 [58.7, 76.2]	78.7 [74.9, 82.0]	0.73
**Metamyl**	296	107	35	45	86.8 [82.9, 90.1]	75.4 [67.9, 81.7]	89.4 [85.5, 92.4]	70.4 [62.5, 77.2]	83.4 [79.5, 86.3]	1.00
**Pafig**	331	68	74	10	97.1 [94.8, 98.6]	47.9 [40.2, 56.3]	81.7 [77.3, 85.3]	87.2 [78.4, 93.8]	82.6 [78.7, 85.7]	1.00
**Pasta**	224	124	18	117	65.7 [60.8, 70.6]	87.3 [81.1, 91.9]	92.6 [88.6, 95.3]	51.5 [45.2, 57.9]	72.0 [67.7, 75.6]	0.03
**Pasta2**	208	129	13	133	61.0 [55.7, 65.7]	90.8 [85.1, 94.7]	94.1 [90.5, 96.8]	49.2 [43.2, 55.6]	69.8 [65.4, 73.5]	2.32E-03
**Tango**	191	132	10	150	56.0 [50.4, 60.9]	93.0 [87.7, 96.5]	95.0 [91.1, 97.5]	46.8 [41.1, 53.0]	66.9 [62.3, 70.8]	4.07E-05
**Waltz**	168	132	10	173	49.3 [43.8, 54.5]	93.0 [87.7, 96.5]	94.4 [90.2, 97.1]	43.3 [37.7, 49.2]	62.1 [57.3, 66.0]	2.42E-07
**Zyggregator**	228	116	26	113	66.9 [62.0, 72.0]	81.7 [74.8, 87.5]	89.8 [85.4, 93.0]	50.7 [44.4, 57.5]	71.2 [67.2, 75.4]	0.01

## Discussion

### Orthogonal based artificial neural networks

The orthogonal encoding of the amino acids, due to its simplicity, has been used in several secondary structure prediction algorithms [45,60,61], even if only to establish an internal reference to compare to the developed predictor and determine if the features included are more informative than merely, the type of amino acids present within the sequence and their relative position [62,63]. With this in mind, several artificial neural networks have been trained, based on input vectors computed through the orthogonal encoding of amino acids, from which, the neural network with the best overall accuracy and lowest accuracy differences between training, testing and validation sub-dataset classifications was selected, as described in the methods section. This neural network showed relatively high values of sensitivity (83.0%), specificity (82.6%), positive and negative predictive values (80.0% and 85.3% respectively) and overall accuracy (82.8%) in the classification of the sequences present in the training sequences dataset. However, the results obtained for the classification of the sequences present in the external validation sequences dataset were, except for sensitivity (90.6%), considerably lower for specificity (31.0%), positive and negative predictive values (75.9% and 57.9% respectively) and overall accuracy (73.1%). These results could be an indication that the rules developed by the selected neural network are not easily generalized across more diverse sequences or that the relationships established between the features present in the input vectors and expected outcomes do not entirely describe the amyloid forming propensity problem when transposed to peptide and protein sequences with lengths greater than six amino acids. It is interesting however, that this encoding method allowed amyloidogenicity propensity prediction with relatively high accuracy (73.1%), considering the simplicity of the information provided to the learning algorithm.

### Physicochemical and biochemical based neural networks

The physicochemical and biochemical description of the polypeptide sequences was obtained through the encoding sequence based on the APDBase and AAindex databases of physicochemical and biochemical properties of the amino acids. After which, feature selection was performed with the purpose of reducing the dimensionality of the computed input vectors, enabling the smallest subset of features to be chosen while providing the highest possible generalization, so improving the neural networks’ learning performance. In the present study, feature selection was performed using three wrapper methods of recursive feature selection, caret, Boruta, and penalizedSVM with five different classifiers (spls, sda, nb for caret, rf for Boruta and svm for penalizedSVM). This resulted in the selection of 96, 10, 13, 548 and 810 features for the input vectors computed through the APDBase encoding database and 109, 14, 334, 100 and 969 features for the input vectors computed through the AAindex encoding dataset for rf, nb, svm, sda and spls, respectively. For each subset of selected features, new input vectors were created and used in the training of several neural networks from which 5 neural networks per encoding dataset were selected. The selected neural networks showed high values of sensitivity (73.5% to 87.9%), specificity (70.2% to 95.6%), positive and negative predictive values (71.2% to 94.9% and 78.9% to 90.3% respectively) and overall accuracy (76.7% to 91.9%) in the classification of the sequences present in the training sequences dataset. However, in the classification of the sequences present in the external validation sequences dataset, only three of the selected neural networks (NN_APD_rf, NN_AA_rf and NN_AA_nb in Table 2) for both encoding schemes, showed high values of sensitivity (89.4%, 90.8% and 87.4%), specificity (67.8%, 62.6% and 78.9%), positive (86.8%, 85.3% and 90.8%) and negative (73.5%, 73.4% and 72.3%) predictive values and overall accuracy (82.2% and 84.9%), suggesting that svm, sda and spls were not the most appropriate classifier methods for this specific type of classification problem in contrast to rf and nb classifiers.

### Analysis of the amyloidogenicity propensity prediction of the selected artificial neural network

The artificial neural network based on the description of the polypeptide sequences through the physicochemical and biochemical properties of the amino acids that showed the highest overall accuracy, and therefore, the most successful predictor developed, was selected for further study (NN_AA_nb). This neural network, henceforth referred as APPNN, showed values of sensitivity, specificity, positive predictive value, negative predictive value and overall accuracy of 87.4%, 70.2%, 71.1%, 86.9% and 78.0%, respectively, when classifying the sequences present in the training sequences dataset and 87.4%, 78.9%, 90.9%, 72.3% and 84.9%, respectively, when classifying sequences present in the external validation sequences dataset.

This newly developed predictor was generated from training with input vectors computed through the AAindex database of physicochemical and biochemical properties of amino acids, after recursive feature selection with the internal classifier Naïve Bayes, where a subset of 14 features was selected. These were the summation of the values of the Normalized frequency of β-sheet [43], the Normalized frequency of β-sheet from a dataset of 44 sample proteins named LG [44], the weights of a first order neural network neuronal for β-sheet at the window position of 1 [45], the standard deviation of the isoelectric point [46], and standard deviation, range and mean absolute deviation of the atom-based hydrophobic moment [47], the helix termination parameter or theoretical estimate of helix-coil stability parameter for the natural occurring amino acids when found at position j+1 of the C-terminal region of the helix [48], and the ΔG° values, which provides a measure of structural stability, for the peptides extrapolated to 0 M of urea from a two-state model derived from urea denaturation curves that correlated the dissociation constants of the peptides containing one of the 20 natural occurring amino acids in a guest position, with the urea concentration [49]. Interestingly the amino acids’ propensity to form β-sheet and α-helices and the hydrophobic moment have been consistently pointed out in the literature as fundamental factors in the molecular mechanism of amyloid formation and are proved here to have a high correlation with the ability of a sequence to form amyloid [7,37,50–53]. Moreover, the isoelectric point of the amino acids in a peptide or protein sequence can affect the intrinsic propensity of a sequence to undergo conformational changes into amyloid fibrils as a result of charge variations caused by pH changes in the environment. The ΔG° values for peptides extrapolated to 0 M of urea is a quantitative measure of the effect of the amino acid point mutations on the conformational stability of peptides. This can be directly correlated with the propensity for a peptide or protein to undergo conformational changes into amyloid intermediates and consequently amyloid fibers, as polypeptides generally need to partially fold (intrinsically unfolded polypeptides) or partially unfold (globular proteins) in order to achieve the β-rich amyloidogenic intermediates [64]. Interestingly, these features fit into the three major groups of features identified by Maurer-Stroh and co-workers in the development of the Waltz algorithm (α-helical, β-sheet and solvation-related hydrophobicity propensities).

A more detailed analysis of the APPNN classification results obtained using training and external validation sequences datasets, showed some level of disagreement in terms of sensitivity, specificity, positive and negative predictive values and accuracy. This was found to be even more marked for the neural networks that had been developed using feature selection based on other methodologies. This could be due to the low number of training sequences, which allied to the low variability of these sequences and may have led to an small over fitting of the neural network and thus to a lower generalization capability when classifying never seen sequences. Moreover, the assumption that for a sequence to be considered amyloidogenic there needs to be at least one amyloidogenic six amino acid stretch within the sequence (hard coded into the algorithm), does not take into account the fact that only one stretch may not be enough to effectively produce the destabilization of the entire peptide or protein structure required for the transition from its native state into amyloid fibers. Additionally, it does not take into account possible interactions between stretches, amyloidogenic or non-amyloidogenic, which could enhance or even inhibit amyloid formation. For these reasons it is thus expected that the length of the input sequences may play a major role in prediction results, where the overall accuracy of our predictor should be higher for smaller sequences. In order to mitigate this problem, the algorithm developed here provides a per amino acid prediction score, which could be used for further analysis.

A comparative analysis between the accuracy of APPNN and several others published prediction methods (Aggrescan, AMYLPRED, AMYLPRED2, FoldAmyloid, MetAmyl, Pafig, Pasta, Pasta2, Tango, Waltz and Zyggregator), was undertaken for the classification of sequence datasets assembled from the literature (training sequences and external validation sequences datasets). The results show that APPNN has high overall accuracies for the classification of the training (78.0%) and the external validation (84.9%) sequence datasets. MetAmyl outperformed APPNN in the classification of the training sequences dataset with an accuracy of 79.1%, although APPNN outperformed MetAmyl in the classification of the external validation sequences dataset, where MetAmyl showed an accuracy value of 83.4%. Analysis showed that the differences between the accuracy values obtained for APPNN and the other predictions was statistically significant hence confirming that the APPNN was able to provide enhanced propensity prediction compared to Pasta (p-value of 0.03), Pasta2 (p-value of 2.32E-03) Tango (p-value of 4.07E-05), Waltz (p-value of 2.42E-07) and Zyggregator (p-value of 0.01).

In this study we have thus developed a highly accurate and effective method for the prediction of amyloid propensity based on the polypeptide amino acid sequence alone. This was achieved using a very small subset of highly relevant physicochemical and biochemical amino acid properties. Overall, this study not only provides a new amyloidogenicity propensity prediction method but also gives new insights into the key driving forces underpinning the self-assembly of peptides and proteins into amyloid-like fibers.

## Methods

### Sequence datasets

A dataset of polypeptide sequences with experimental *in vitro* evidence of amyloid formation was assembled from the combination of sequences present in published datasets used in several amyloidogenicity propensity prediction studies [28,29,33,34,54,55,65–68]. In addition, several sequences meeting this requirement were also added (see S1 and S2 files for references). This resulted in the construction of two distinct datasets (training sequences dataset and external validation sequences dataset) in which each sequence is associated with a binary target value, representing its ability to form amyloid. The training sequences dataset (S1 File) is exclusively formed by peptides of six amino acids in length, with a total of 296 sequences, from which 161 have been reported negatively and 125 positively for amyloid formation. The external validation sequences dataset (S2 File) is a more general dataset comprising a total of 483 peptide and protein sequences with lengths greater than six amino acids, from which 142 have been reported negatively and 341 positively for amyloid formation.

### Sequence encoding

In order to convert sequence information into numerical vectors that could identify each sequence uniquely and be used to train the artificial neural networks, three encoding schemes were prepared in MATLAB [69]. These schemes utilized a simple orthogonal encoding for the twenty naturally occurring amino acids, two datasets of amino acid physicochemical and biochemical properties, the Amino Acid Index Database version 9.1 (AAindex) [58,59] and the Amino Acid Physicochemical Properties Database (APDBase) [57].

AAindex is a database of numerical indices that represent the physicochemical and biochemical properties of individual amino acids, from which only the first dataset (aaindex1) was used. This dataset contains a total of 544 characteristics, 531 of which, had no missing values for any of the twenty natural occurring amino acids and thus were used in the calculations [58,59].

APDBase is a smaller database containing a total of 242 physicochemical and biochemical properties for all twenty naturally occurring amino acids. APDBase was derived from two other databases, AAindex and ProtScale by Mathura and Kolippakkam, based on properties they felt were most relevant to the study of protein sequence, structure, and function [57].

Sequences present in both training and external validation sequence datasets were encoded through *in house* built scripts in MATLAB [69] programing language. Feature vectors based on the orthogonal descriptors of the amino acids, were created by the linear combination of the respective individual amino acids orthogonal vectors (S1 Table). Feature vectors, based on the physicochemical and biochemical properties of the amino acids were obtained by the concatenation of several smaller vectors containing the single characteristics of the amino acids, the cumulative summation of these characteristics and some basic mathematical and statistical measures of these characteristics (summation, mean, harmonic mean, median, mode, standard deviation, interquartile range, mean absolute deviation, range, kurtosis and skewness).

### Feature vectors pre-processing

Pre-processing was performed for all generated feature vectors, prior to training and external validation of the neural networks, through data normalization by mapping the mean and standard deviation to 0 and 1 respectively (except for feature vectors based on the orthogonal encoding), through removal of features with no variation across samples and removal of duplicated features. This pre-processing was performed to circumvent scale effects of some features over others and to improve feature selection performance.

### Feature selection

Feature selection was performed for the characteristics vectors computed through both physicochemical and biochemical properties of the amino acids encoding datasets (AAindex and APDBase). Feature selection was performed utilizing three recursive feature selection wrapper methods, from the caret package v.5.15–48 [70], Boruta package v.1.6 [71] and penalizedSVM package v.1.1 [72] for R v.2.15.3 [73] with four different internal classifiers (sparse partial least squares (spls), shrinkage discriminant analysis (sda), both linear, and naïve bayes (nb) for caret, and random forests (rf) for Boruta and support vector machines (svm) for penalizedSVM). After this the input vectors were trimmed to match each set of selected features and subsequently used in the training of 5 neural networks per encoding dataset. The generated neural networks were posteriorly validated through the classification of the external validation sequences dataset as described below.

### Artificial neural networks

MATLAB’s [69] Neural Networks Toolbox [74] was used to create feed forward fully connected neural networks. The weights and biases of the neural networks were initialized with the Nguyen-Widrow layer initialization function (initializing weights and biases randomly but evenly across each layer’s input space). The activation function selected for the hidden layer was the symmetric sigmoid function and for the output layer was the linear function. The learning algorithm used was the scaled conjugate gradient backpropagation (backward propagation of errors) and the performance measure used to stop training was the mean absolute error. The number of neurons present in the input layer was set to match the dimensions of the different feature vectors. The number of neurons present in the hidden layer was computed based on the number of dimensions of the feature vectors (_*n/3*_ *)* for the orthogonal based vectors and _*(n/2 +1)*_ for the physicochemical and biochemical properties of the amino acid based vectors). The number of neurons in the output layer was one.

For each of the computed input vectors, neural networks were trained after random division of the input sequences dataset (the hexapeptides dataset) into three distinct subsets, the training, test and validation subsets, comprising 70%, 15% and 15% of the overall training samples, respectively. The best neural network was selected for the feature vectors computed through the orthogonal encoding scheme from a total of 5000 trained networks, and for feature vectors computed through the physicochemical and biochemical encoding scheme from a total of 1000 trained networks. This selection was based on the values of accuracy and standard deviation obtained for the training, test, validation subsets and overall dataset, where the neural network with the highest average accuracy was selected, provided that the standard deviation was below 7.5%. The selected neural networks were posteriorly validated by the classification of the sequences present in the external validation dataset. This was performed by the submission of the pre-processed individual input vectors, generated by a sliding window of six amino acids that was run through the polypeptide sequence, to the corresponding neural network. A sequence was considered amyloidogenic if at least one of these six amino acid windows was classified amyloidogenic.

### Comparison with other prediction algorithms

A careful comparison of the best neural network (APPNN) and other published methods for amyloid propensity prediction (Aggrescan, AMYLPRED, AMYLPRED2, FoldAmyloid, MetAmyl, Pafig, Pasta, Tango, Waltz and Zyggregator) was undertaken. This comparison took into account that both training sequences and external sequences validation datasets have been produced from the literature specifically for this work. Thus the amyloidogenic propensities of the sequences present in both datasets were evaluated by all these methods through the use of *in house* built Python version 2.7.5 [75] scripts that allowed sequence submission and results retrieval (input parameters and considerations made to classify a sequence as amyloidogenic from the provided outputs, can be found in S2 Table, organized by prediction method).

Prediction results for both training sequences and external validation sequence datasets were summarized for each predictor in confusion matrices containing the obtained values for sensitivity, specificity, positive predictive value, negative predictive value and accuracy, with corresponding 95% confidence intervals. These values were calculated with the R v.2.15.1 [73] package boot v.1.2–10 [76,77] through bootstrapping performed with 2000 replicates. Moreover, after 10-fold stratified resampling of the data with the package caret v.5.15–48 [70], the Friedman’s test provided in the package coin v.1.0–23 [78,79] was used to determine if the accuracy values obtained for all predictors were sampled from populations with identical distributions, which was followed by the Wilcoxon-Nemenyi-McDonald-Thompson post-hoc test [80] from the package multcmp v.1.3–2 [81], for pairwise comparison between APPNN and every other classifier.

## Supporting Information

S1 File Training sequences dataset. (DOCX)

S2 File External validation sequences dataset. (DOCX)

S1 Table Orthogonal encoding of the amino acids. (DOCX)

S2 Table Input parameters and considerations made to classify a sequence as amyloidogenic from the provided outputs, organized by prediction method. (DOCX)
