# Supplementary material for: Prediction of Peptide and Protein Propensity for Amyloid Formation
Source: PLoS One. 2015 Aug 4;10(8):e0134679. doi: 10.1371/journal.pone.0134679 (PMC4524629; doi:10.1371/journal.pone.0134679)
Supplement: S1 File — (DOCX) [file pone.0134679.s001.docx]

# S1 File. Training sequences dataset.

| **Sequence** | **Amyloid** | **References** |
| --- | --- | --- |
| AAELRN | 0 | 1 |
| AARRFF | 0 | 1 |
| AEKLFD | 0 | 1 |
| AEMEYL | 0 | 1 |
| AENGKS | 0 | 1, 2 |
| AEVLAL | 0 | 1 |
| AGVNYF | 1 | 1 |
| AINKIQ | 0 | 1 |
| ALEEYT | 1 | 1 |
| AQFIIS | 1 | 1 |
| ASSSNY | 1 | 5 |
| ATVIIE | 1 | 1, 2 |
| DADLYL | 0 | 1 |
| DAVKIS | 0 | 6 |
| DCVNIT | 1 | 1 |
| DDSLFF | 0 | 1 |
| DECFFF | 0 | 1 |
| DIEVDL | 0 | 1, 2 |
| DILTYT | 0 | 1 |
| DLLKNG | 0 | 1, 2 |
| DTVIIE | 1 | 1, 2 |
| EALYLV | 0 | 1, 2 |
| ECFFFE | 0 | 1 |
| EECLFL | 0 | 1 |
| EGVLYV | 1 | 1 |
| EHSDLS | 0 | 1, 2 |
| EIPAGV | 0 | 6 |
| EKDEYA | 0 | 1, 2 |
| EKNLYL | 0 | 1 |
| ERGFFY | 0 | 1, 2 |
| ERIEKV | 0 | 1, 2 |
| ETVIIE | 1 | 1, 2 |
| ETWFFG | 0 | 1 |
| ETYVVT | 0 | 6 |
| EVDLLK | 0 | 1, 2 |
| EYLKIA | 0 | 1 |
| EYSNFS | 1 | 1 |
| FERQHM | 0 | 5 |
| FESNFN | 1 | 1 |
| **Sequence** | **Amyloid** | **References** |
| FFWRFM | 0 | 1 |
| FFYTPK | 0 | 1, 2 |
| FGELFE | 0 | 1 |
| FHPSDI | 0 | 1, 2 |
| FLKYFT | 0 | 1 |
| FLVHSS | 1 | 1, 2 |
| FMFFII | 0 | 1 |
| FSKDWS | 0 | 1, 2 |
| FTPTEK | 0 | 1, 2 |
| FTVIIE | 1 | 1, 2 |
| FVNQHL | 0 | 1, 2 |
| FYLLYY | 1 | 1, 2 |
| FYTPKT | 0 | 1, 2 |
| GERGFF | 0 | 1, 2 |
| GEWTYD | 1 | 1 |
| GFFYTP | 0 | 1, 2 |
| GGVVIA | 1 | 5 |
| GMFNIQ | 0 | 1 |
| GNTCVN | 1 | 1 |
| GNVCIN | 1 | 1 |
| GSHLVE | 0 | 1, 2 |
| GTFFIN | 1 | 1 |
| GTFFIT | 1 | 1 |
| GTFNII | 1 | 1 |
| GTVIIE | 1 | 1, 2 |
| GTVLFM | 1 | 1 |
| GVNYFL | 1 | 1 |
| HAFLII | 0 | 1 |
| HFVWIA | 1 | 1 |
| HGWLIM | 1 | 1 |
| HIFIIM | 0 | 1 |
| HKALFW | 0 | 1 |
| HLVEAL | 0 | 1, 2 |
| HLVYIM | 1 | 1 |
| HPAENG | 0 | 1, 2 |
| HQLIIM | 1 | 1 |
| HSSNNF | 1 | 5 |
| HYFNIF | 1 | 1 |
| ICSLYQ | 0 | 1 |
| **Sequence** | **Amyloid** | **References** |
| IEKVEH | 0 | 1, 2 |
| IEMIFV | 1 | 1 |
| IFDFIQ | 1 | 1 |
| IHKAQN | 0 | 5 |
| ILENIS | 0 | 1 |
| IQRTPK | 0 | 1, 2 |
| IQVYSR | 0 | 1, 2 |
| ISFLIF | 1 | 1 |
| ISMTTS | 0 | 5 |
| ITVIIE | 1 | 1, 2 |
| KAFIIQ | 0 | 1 |
| KAILFL | 0 | 1 |
| KDWSFY | 1 | 1, 2 |
| KECLIN | 0 | 1 |
| KENIIF | 0 | 1 |
| KGENFT | 0 | 1 |
| KHIIVA | 1 | 5 |
| KIVKWD | 1 | 1, 2 |
| KLFIIQ | 1 | 1 |
| KLLEIA | 0 | 1 |
| KLLIYE | 1 | 1 |
| KMFFIQ | 0 | 1 |
| KSHPET | 0 | 6 |
| KSNFLN | 0 | 1, 2 |
| KTVIIE | 0 | 1, 2 |
| KTVIIT | 0 | 1, 2 |
| KTVIVE | 0 | 1, 2 |
| KTVIYE | 0 | 1, 2 |
| KTVLIE | 0 | 1, 2 |
| KVEHSD | 0 | 1, 2 |
| KVQIIN | 1 | 1 |
| KWDRDM | 0 | 1, 2 |
| LAEAIG | 0 | 1 |
| LAVLFL | 1 | 1 |
| LIAGFN | 0 | 1, 2 |
| LIFLIV | 1 | 1 |
| LKNGER | 0 | 1, 2 |
| LLYYTE | 1 | 1, 2 |
| LMSLFG | 0 | 1 |
| **Sequence** | **Amyloid** | **References** |
| LSFSKD | 0 | 1, 2 |
| LSQPKI | 0 | 1, 2 |
| LTVIIE | 1 | 1, 2 |
| LVEALY | 1 | 1, 2 |
| LYQLEN | 1 | 1, 2, 5 |
| MIENIQ | 1 | 1 |
| MLVLFV | 1 | 1 |
| MTVIIE | 1 | 1, 2 |
| MVGGVV | 1 | 5 |
| MYFFIF | 0 | 1 |
| MYWIIF | 0 | 1 |
| NFGAIL | 1 | 1, 2, 3, 4, 8, 9 |
| NFGSVQ | 1 | 7, 10 |
| NFLVHS | 1 | 5 |
| NGERIE | 0 | 1, 2 |
| NGKSNF | 0 | 1, 2 |
| NHVTLS | 1 | 1, 2 |
| NIQYQF | 0 | 1 |
| NIVLIM | 0 | 1 |
| NLGPVL | 0 | 1, 2 |
| NNQNTF | 1 | 5 |
| NNQQNY | 1 | 5 |
| NQFIIS | 1 | 1 |
| NQQNQY | 1 | 1 |
| NTVIIE | 1 | 1, 2 |
| NYFAIR | 0 | 1 |
| NYNTYR | 0 | 1 |
| NYVWIV | 1 | 1 |
| PKIQVY | 0 | 1, 2 |
| PSDIEV | 0 | 1, 2 |
| PTEKDE | 0 | 1, 2 |
| PTVIIE | 0 | 1, 2 |
| QANKHI | 1 | 5 |
| QAQNQW | 0 | 1 |
| QEMRHF | 1 | 5 |
| QLENYC | 1 | 1 |
| QPKIVK | 0 | 1, 2 |
| QTNLYG | 0 | 1 |
| QTVIIE | 1 | 1, 2 |
| RETWFF | 0 | 1 |
| RGFFYT | 0 | 1, 2 |
| RLVFID | 0 | 1 |
| **Sequence** | **Amyloid** | **References** |
| RMFNII | 0 | 1 |
| RNLTKD | 0 | 5 |
| RQVLIF | 1 | 1 |
| RRWQWR | 1 | 11 |
| RTPKIQ | 0 | 1, 2 |
| RVAFFE | 0 | 1 |
| RVFNIM | 1 | 1 |
| RVNHVT | 0 | 1, 2 |
| SAVIIE | 1 | 1, 2 |
| SDLSFS | 0 | 1, 2 |
| SDVIIE | 1 | 1, 2 |
| SEVIIE | 1 | 1, 2 |
| SFFFIQ | 1 | 1 |
| SFLIFL | 1 | 1 |
| SFQIYA | 1 | 1 |
| SFVIIE | 1 | 1, 2 |
| SGDGSL | 0 | 6 |
| SGFHPS | 0 | 1, 2 |
| SGVIIE | 1 | 1, 2 |
| SHLVEA | 0 | 1, 2 |
| SIVIIE | 1 | 1, 2 |
| SLVIIE | 1 | 1, 2 |
| SLYQLE | 0 | 1, 2 |
| SMVIIE | 1 | 1, 2 |
| SMVLFS | 1 | 1 |
| SNQNNF | 1 | 5 |
| SNVIIE | 1 | 1, 2 |
| SPVIIE | 0 | 1, 2 |
| SQFYIT | 1 | 1 |
| SQVIIE | 1 | 1, 2 |
| SRHPAE | 0 | 1, 2 |
| SSNNFG | 0 | 1 |
| SSTNVG | 1 | 5 |
| SSTSAA | 1 | 5 |
| SSVIIE | 1 | 1, 2 |
| STAIIE | 0 | 1, 2 |
| STDIIE | 0 | 1, 2 |
| STEIIE | 1 | 1, 2 |
| STFIIE | 1 | 1, 2 |
| STGIIE | 0 | 1, 2 |
| STIIIE | 0 | 1, 2 |
| STLIIE | 1 | 1, 2 |
| **Sequence** | **Amyloid** | **References** |
| STMIIE | 0 | 1, 2 |
| STMSIT | 1 | 5 |
| STNIIE | 1 | 1, 2 |
| STPIIE | 0 | 1, 2 |
| STQIIE | 1 | 1, 2 |
| STSIIE | 1 | 1, 2 |
| STTIIE | 0 | 1, 2 |
| STVAIE | 0 | 1, 2 |
| STVDIE | 0 | 1, 2 |
| STVEIE | 1 | 1, 2 |
| STVFIE | 1 | 1, 2 |
| STVGIE | 0 | 1, 2 |
| STVIAE | 0 | 1, 2 |
| STVIDE | 0 | 1, 2 |
| STVIEE | 0 | 1, 2 |
| STVIFE | 1 | 1, 2 |
| STVIGE | 0 | 1, 2 |
| STVIIA | 1 | 1, 2 |
| STVIID | 1 | 1, 2 |
| STVIIE | 1 | 1, 2 |
| STVIIF | 1 | 1, 2 |
| STVIII | 1 | 1, 2 |
| STVIIL | 1 | 1, 2 |
| STVIIM | 1 | 1, 2 |
| STVIIN | 1 | 1, 2 |
| STVIIP | 0 | 1, 2 |
| STVIIQ | 1 | 1, 2 |
| STVIIS | 1 | 1, 2 |
| STVIIT | 1 | 1, 2 |
| STVIIV | 1 | 1, 2 |
| STVIIW | 1 | 1, 2 |
| STVIIY | 1 | 1, 2 |
| STVILE | 0 | 1, 2 |
| STVIME | 0 | 1, 2 |
| STVINE | 0 | 1, 2 |
| STVIPE | 0 | 1, 2 |
| STVIQE | 0 | 1, 2 |
| STVISE | 0 | 1, 2 |
| STVITE | 0 | 1, 2 |
| STVIVE | 0 | 1, 2 |
| STVIWE | 0 | 1, 2 |
| STVIYE | 1 | 1, 2 |
| **Sequence** | **Amyloid** | **References** |
| STVLIE | 1 | 1, 2 |
| STVMIE | 0 | 1, 2 |
| STVNIE | 1 | 1, 2 |
| STVPIE | 0 | 1, 2 |
| STVQIE | 0 | 1, 2 |
| STVSIE | 0 | 1, 2 |
| STVTIE | 1 | 1, 2 |
| STVVIE | 0 | 1, 2 |
| STVWIE | 1 | 1, 2 |
| STVYIE | 1 | 1, 2 |
| STYIIE | 0 | 1, 2 |
| SVVIIE | 1 | 1, 2 |
| SWVIIE | 0 | 1, 2 |
| SYSTMS | 1 | 5 |
| SYVIIE | 1 | 1, 2 |
| TAELIT | 0 | 1 |
| TAWYAE | 0 | 1 |
| TEFTPT | 0 | 1, 2 |
| TFQINS | 1 | 5 |
| TFWEIS | 0 | 1 |
| TLKNYI | 0 | 1 |
| **Sequence** | **Amyloid** | **References** |
| TNELYM | 0 | 1 |
| TTVIIE | 1 | 1, 2 |
| TYQIIR | 0 | 1 |
| TYSFYC | 0 | 6 |
| TYVEYI | 1 | 1 |
| VAWLKM | 0 | 1 |
| VEALYL | 1 | 1, 2, 5 |
| VILLIS | 1 | 1 |
| VKWDRD | 0 | 1, 2 |
| VQIVYK | 1 | 1, 2, 5, 6, 7 |
| VSFEIV | 1 | 1 |
| VTLSQP | 0 | 1, 2 |
| VTQEFW | 0 | 1 |
| VTSTFS | 1 | 1 |
| VTVIIE | 1 | 1, 2 |
| VYSRHP | 0 | 1, 2 |
| WIVIFF | 1 | 1 |
| WSFYLL | 0 | 1, 2 |
| WTVIIE | 1 | 1, 2 |
| WVENYP | 0 | 1 |
| WVFWIG | 1 | 1 |
| **Sequence** | **Amyloid** | **References** |
| WYFYIQ | 0 | 1 |
| YASEIE | 0 | 1 |
| YLEIII | 0 | 1 |
| YLNWYQ | 1 | 1 |
| YQLENY | 0 | 1, 2 |
| YTFTIS | 0 | 1 |
| YTVIIE | 1 | 1, 2 |
| YVEYIG | 1 | 1 |
| YVSGFH | 0 | 1, 2 |
| YYQNYQ | 1 | 1 |
| YYTEFT | 0 | 1, 2 |
| AAELRN | 0 | 1 |
| AARRFF | 0 | 1 |
| AEKLFD | 0 | 1 |
| AEMEYL | 0 | 1 |
| AENGKS | 0 | 1, 2 |
| AEVLAL | 0 | 1 |
| AGVNYF | 1 | 1 |
| AINKIQ | 0 | 1 |

**Bibliography**

1. S. Maurer-Stroh, M. Debulpaep, N. Kuemmerer, M. Lopez de la Paz, I. C. Martins, J. Reumers, K. L. Morris, A. Copland, L. C. Serpell, L. Serrano, J. W. H. Schymkowitz, and F. Rousseau, “Exploring the sequence determinants of amyloid structure using position-specific scoring matrices.,” Nat. Methods, vol. 7, no. 3, pp. 237–242, Mar. 2010.
2. M. J. Thompson, S. a Sievers, J. Karanicolas, M. I. Ivanova, D. Baker, and D. Eisenberg, “The 3D profile method for identifying fibril-forming segments of proteins.,” in Proceedings of the National Academy of Sciences of the United States of America, 2006, vol. 103, no. 11, pp. 4074–4078.
3. O. Conchillo-Solé, N. S. de Groot, F. X. Avilés, J. Vendrell, X. Daura, and S. Ventura, “AGGRESCAN: a server for the prediction and evaluation of ‘hot spots’ of aggregation in polypeptides.,” BMC Bioinformatics, vol. 8, no. 65, pp. 1–17, Jan. 2007.
4. Y. Mazor, S. Gilead, I. Benhar, and E. Gazit, “Identification and characterization of a novel molecular-recognition and self-assembly domain within the islet amyloid polypeptide.,” J. Mol. Biol., vol. 322, no. 5, pp. 1013–1024, Oct. 2002.
5. L. Goldschmidt, P. K. Teng, R. Riek, and D. Eisenberg, “Identifying the amylome, proteins capable of forming amyloid-like fibrils.,” Proc. Natl. Acad. Sci. U. S. A., vol. 107, no. 8, pp. 3487–3892, Feb. 2010.
6. A.-M. Fernandez-Escamilla, F. Rousseau, J. W. H. Schymkowitz, and L. Serrano, “Prediction of sequence-dependent and mutational effects on the aggregation of peptides and proteins.,” Nat. Biotechnol., vol. 22, no. 10, pp. 1302–1306, Oct. 2004.
7. J. Tian, N. Wu, J. Guo, and Y. Fan, “Prediction of amyloid fibril-forming segments based on a support vector machine.,” BMC Bioinformatics, vol. 10 Suppl 1, no. S45, pp. 1–8, Jan. 2009.
8. L. Miravalle, T. Tokuda, R. Chiarle, G. Giaccone, O. Bugiani, F. Tagliavini, B. Frangione, and J. Ghiso, “Substitutions at codon 22 of Alzheimer’s abeta peptide induce diverse conformational changes and apoptotic effects in human cerebral endothelial cells.,” J. Biol. Chem., vol. 275, no. 35, pp. 27110–6, Sep. 2000.
9. R. Azriel and E. Gazit, “Analysis of the minimal amyloid-forming fragment of the islet amyloid polypeptide. An experimental support for the key role of the phenylalanine residue in amyloid formation.,” J. Biol. Chem., vol. 276, no. 36, pp. 34156–61, Sep. 2001.
10. S. Gilead and E. Gazit, “Self-organization of Short Peptide Fragments: From Amyloid Fibrils to Nanoscale Supramolecular Assemblies,” Supramol. Chem., vol. 17, no. 1–2, pp. 87–92, Jan. 2005.
11. S. Farnaud and R. W. Evans, “Lactoferrin - A multifunctional protein with antimicrobial properties.,” Mol. Immunol., vol. 40, no. 7, pp. 395–405, Nov. 2003.
