# Supplementary material for: Prediction of Peptide and Protein Propensity for Amyloid Formation
Source: PLoS One. 2015 Aug 4;10(8):e0134679. doi: 10.1371/journal.pone.0134679 (PMC4524629; doi:10.1371/journal.pone.0134679)
Supplement: S2 File — (DOCX) [file pone.0134679.s002.docx]

## S2 File. External validation sequences dataset

| **Sequence** | **Amyloid** | **References** |
| --- | --- | --- |
| AAAKAALNAVLVGANA | 1 | 1 |
| ADLPAPDDTGLQAVLHTALSQGAPGAMVRVDDNGTIHQLSEGVADRATGRAITTTDRFRVGSVTKSFSAVVLLQLVDEGKLDLDASVNTYLPGLLPDDRITVRQVMSHRSGLYDYTNDMFAQTVPGFESVRNKVFSYQDLITLSLKHGVTNAPGAAYSYSNTNFVVAGMLIEKLTGHSVATEYQNRIFTPLNLTDTFYVHPDTVIPGTHANGYLTPDEAGGALVDSTEQTVSWAQSAGAVISSTQDLDTFFSALMSGQLMSAAQLAQMQQWTTVNSTQGYGLGLRRRDLSCGISVYGHTGTVQGYYTYAFASKDGKRSVTALANTSNNVNVLNTMARTLESAFCGKPTT | 1 | 2, 3 |
| AEFHRWSSYMVHWK | 1 | 2, 4, 5 |
| AEFHRWSSYMVYWK | 1 | 6, 7 |
| AEGYQYRALYDYKKEREEDIDLHLGDILTVNKGSLVALGFSDGQEARPEEIGWLNGYNETTGERGDFPGTYVEYIGR | 1 | 8 |
| AEQLLQEAEQLLQEL | 1 | 9 |
| AESDKKEEEK | 0 | 10 |
| AEVSIVV | 1 | 10 |
| AFGAILSS | 1 | 6, 7, 11 |
| AGAAAAGAVVGGLGG | 1 | 6, 7 |
| AGFHRWNNYMMDWK | 0 | 2, 5 |
| AGYQQQYNPQ | 0 | 10 |
| AIAAIVF | 1 | 10 |
| AIIGLMVGGVV | 1 | 3, 12 |
| AKKENIIAAAQAGASGY | 1 | 7, 13 |
| AKNVDYCKEWLVNHIK | 0 | 13 |
| ALNEINQFYQKFPQYLQYLYQGPIVLNPWDQVKRNAVPITPTLNR | 1 | 12 |
| ALWDTLLKKVLKAAAKAALDAVLVGANA | 1 | 1 |
| ALWDTLLKKVLKAAAKAALNAVLVGANA | 1 | 1 |
| ALWKNMLKGIGKLAGKAALGAVKKLVGAES | 1 | 14 |
| ALWKTLLKKV | 1 | 1 |
| ALWKTLLKKVLKA | 1 | 1 |
| ALWKTLLKKVLKAAA | 1 | 1 |
| ALWKTLLKKVLKAAAK | 1 | 1 |
| ALWKTLLKKVLKAAAKAALKAVLVGANA | 1 | 1 |
| ALWKTLLKKVLKAAAKAALNAVLVGANA | 1 | 1 |
| ALWMTLLKKVLK | 1 | 1 |
| ALWMTLLKKVLKAAAK | 1 | 1 |
| ALWMTLLKKVLKAAAKAALDAVLVGANA | 1 | 1 |
| ALWMTLLKKVLKAAAKAALKAVLVGANA | 1 | 1 |
| ALWMTLLKKVLKAAAKAALN | 1 | 1 |
| ALWMTLLKKVLKAAAKAALNAVLVGANA | 1 | 1, 14, 15 |
| AMALNCDPVWLQYGTKRGKA | 0 | 13 |
| ARIIRYFYNAKAG | 0 | 13 |
| ARTVESRQAQDLARSYGIP | 0 | 13 |
| ASMFEAQLSKEKKMFTLL | 0 | 16 |
| ASSQKKMKEMLAFFTLEL | 1 | 16 |
| ATEVPVSWESFNNGDCFILDLGNNIHQWCGSNSNRYERLKATQVSKGIRDNERSGRAR | 1 | 12 |
| ATKAVCVLKGDGPVQGIINFEQKESNGPVKVWGSIKGLTEGLHGFRVHEFGDNTAGCTSAGPHFNPLSRKHGGPKDEERHVGDLGNVTADKDGVADVSIEDSVISLSGDHCIIGRTLVVHEKADDLGKGGNEESTKTGNAGSRLACGVIGIAQ | 1 | 2 |
| ATKHKIP | 0 | 13 |
| ATKKVGTKPA | 0 | 10 |
| ATQRLANFLVHSS | 1 | 3, 17, 18 |
| AVGKSNLLSRYARNEFSA | 0 | 13 |
| AVSVEEGKALAEEEGLF | 0 | 13 |
| AVVTGVTAVAQKTV | 1 | 12 |
| AYVKKLDSGTGKELVLAL | 0 | 13 |
| AYYDGRRSSAVLFSSPP | 1 | 19, 20 |
| CGNLSTCMLGTYTQDFNKFHTFPQTAIGVGAP | 1 | 21, 22, 23 |
| CPLMVKVLDA | 1 | 7, 24 |
| CPLMVKVLDAV | 1 | 6, 7, 25 |
| DAAKYSEV | 0 | 13 |
| DAEFRHDSGYEVHHQKLVFFAEDVGSNK | 1 | 19, 20, 26 |
| DAEFRHDSGYEVHHQKLVFFAEDVGSNKGAIIGLMVGGVV | 1 | 2, 13, 27, 28, 29, 30, 31 |
| DAEFRHDSGYEVHHQKLVFFAEDVGSNKGAIIGLMVGGVVIA | 1 | 6, 27, 28, 29, 30, 31, 32 |
| DAQAIAL | 1 | 10 |
| DCVNITIKQHTVTT | 1 | 6, 7 |
| DCVNITIKQHTVTTTT | 1 | 7 |
| DEPPQSPWDRVKDLATVYVDVLKDSGRDYVSQFEGSALGKQLNLKLLDNWDSVTSTFSKLREQLGPVTQEFWDNLEKETEGLRQEMSKDLEEV | 1 | 12 |
| DFSTMRRIVRNLLKELGYN | 0 | 13 |
| DHPAVMEGTKTILETDSNLS | 0 | 13 |
| DIKIMERVVEQMCTTQY | 1 | 6, 7 |
| DILTLLNSTNKDWWKVEVND | 1 | 7, 13 |
| DIQKVAGTWY | 1 | 12 |
| DIVMTQSQKFMSTSVGDRVSITCKASQNVGTAVAWYQQKPGQSPKLMIYSASNRYTGVPDRFTGSGSGTDFTLTISNMQSEDLADYFCQQYSSYPLTFGAGTKLELKRADAAPTVSIFPPSSEQLTSGGASVVCFLNNFYPKDINVKWKIDGSERQNGVLNSATDQDSKDSTYSMSSTLTLTKDEYERHNSYTCEATHKTSTSPIVKSFNRNEC | 1 | 2 |
| DKKEEEKSAE | 0 | 10 |
| DQKEAALVDMVNDGVEDLRCKYATLIYT | 0 | 13 |
| DRAVVLSHYIHNLSS | 1 | 12 |
| DSGYEVHHQKLVFFAEDVGSNKG | 1 | 13 |
| DVAGHGQDILIRLFKS | 1 | 13 |
| DVKMMERVVEQMCITQY | 1 | 12 |
| DWSFYLLYYTEFT | 1 | 13, 33 |
| DWSFYLLYYTEFTPTEKDEYA | 1 | 3, 32, 33 |
| DWSFYLLYYTEFTPTGKDEYA | 1 | 6, 7, 13 |
| DWWKVEVNDRQGFVPA | 1 | 7, 13 |
| EASNCFAIRHFENKFAVETLICFNLFLNSQEKHY | 1 | 3, 6, 7, 34 |
| EASNCFAIRHFENKFAVETLICSRTVKKNIIEEN | 1 | 3, 4, 6, 7, 34 |
| EDGVDALNKLQAGGY | 0 | 13 |
| EEEKSAETKE | 0 | 10 |
| EEKPVQTEEK | 0 | 10 |
| EEPVKKEEKP | 0 | 10 |
| EESEFEPFIEEISTKISY | 0 | 13 |
| EFQEICA | 1 | 10 |
| EGEWQLVLHVWAKVEADVAGHGQDILIRLFK | 1 | 7, 13 |
| EGFKTLEEGQAVSFEIVEGNRGPQAANVTKEA | 1 | 12 |
| EGVLYVGSKT | 1 | 12 |
| EGWFSFIGEAVRGAGDMWRAYTDMREANYINADKYFHARGNYDAAQRGPGGVWAAKVISDVREALQRLMGHGAEDSMDDQAANEWGRSGKDPNHFRPKGLPDKY | 1 | 3 |
| EKSELPKVED | 0 | 10 |
| ELKPLAQSHA | 0 | 13 |
| ELLAKSEQAKSMLFTFMK | 1 | 16 |
| ELLNAPGETY | 0 | 13 |
| ELPKVEDLKI | 0 | 10 |
| EPDEAEQDSIEFGKKIANIY | 0 | 13 |
| EPSEQFIKQHDFSSY | 0 | 13 |
| EPTKEPTKVE | 0 | 10 |
| EQVTNVGGAVVTG | 1 | 13 |
| EQVTNVGGAVVTGVTAVA | 1 | 7, 13 |
| ETDIKIMERVVEQM | 1 | 19, 20 |
| ETKEPTKEPT | 0 | 10 |
| ETLKMSMFLEAQFKKSAL | 1 | 16 |
| EVEDYFEEAIRAGLH | 0 | 13 |
| EVHHQKLVFFAEDVG | 1 | 13, 35 |
| EVTGIITQGARNFGSVQFVA | 1 | 36 |
| EVVPHKKMHKDFLEKIGGL | 1 | 7, 13 |
| EWYSFLGEAAQGAWDMWRAYSDMREANYIGADKYFHARGNYDAAQRGPGGAWAAKVISDARENSQRVTDFFRHGNSGHGAEDSKADQEWG | 1 | 3 |
| EYSAMRDQYMRTGEG | 0 | 13 |
| FAKFASEKKLESMTLMLQ | 0 | 16 |
| FHPSDIEVDLLK | 0 | 13 |
| FKCRRWQWR | 1 | 37 |
| FKCRRWQWRMKKLGAPSITCVRRAF | 1 | 38 |
| FKSAEDSMRTSGGA | 0 | 13 |
| FLIFLIVG | 1 | 7, 13 |
| FNGLIVV | 1 | 10 |
| FSMLVAAIQSA | 1 | 12 |
| FSMLVAAIQSAGLTETLN | 1 | 12 |
| FVNVEAVKAFLEAHGIAY | 1 | 7, 13 |
| FVNVQAVKVFLESQGIAY | 1 | 7, 13 |
| FVQWFSKFLGRIL | 1 | 39, 40 |
| GAARCQVTLRDLFDR | 1 | 12 |
| GAVVTGVTAVA | 1 | 2, 3 |
| GDAATAEKVFKQYANDNGVD | 0 | 13 |
| GDGKWMRDFEQRMNGYGSV | 0 | 13 |
| GEKIVFKNNAGFPHNVVFDE | 1 | 13 |
| GETYVVTL | 1 | 7, 13 |
| GEWTYDDATKTFTVTE | 1 | 7, 13 |
| GEYTVDVADKGYTLNIKFAGD | 1 | 7, 13 |
| GGKDWWKVGG | 0 | 13 |
| GGYYQNYQGY | 1 | 10 |
| GIGAVLKVLTTGLPALISWIKRKRQQ | 1 | 41 |
| GIGKFLHSAKKFGKAFVGEIMNS | 1 | 39 |
| GIKLANATKK | 0 | 10 |
| GILNIVV | 1 | 10 |
| GKVQIINKKLDL | 1 | 12 |
| GKVQIVYK | 1 | 7, 13 |
| GLGGYMLGSAMSRPMIHFGNDWEDRYYRENMYRYPNQVYYRPVDQYSNQNNFVHDCVNITIKQHTVTTTTKGENFTETDVKMMERVVEQMCVTQYQKESQAYY | 1 | 3 |
| GLINIAT | 1 | 10 |
| GLRSKIWLWVLLMIWQESNKFKKM | 1 | 42 |
| GLSAPVD | 0 | 13 |
| GLSDGEWQQVLNVWGKVEADIAGHGQEVL | 1 | 12 |
| GLSDGEWQQVLNVWGKVEADIAGHGQEVLIRLFTGHPETLEKFDKFKHLKTEAEMKASEDLKKHGTVVLTALGGILKKKGHHEAELKPLAQSHATKHKIPIKYLEFISDAIIHVLHSKHPGDFGADAQGAMTKALELFRNDIAAKYKELGFQG | 1 | 2 |
| GNLPFLGTAGVAGEFPTA | 1 | 6, 7 |
| GNNQQNY | 1 | 6, 7, 43 |
| GNNQQNYQQY | 1 | 10 |
| GPRLDKQGNFNAWVAGSYGNDQWLQVDLGSSKEVTGIITQGARNFGSVQFVA | 1 | 36 |
| GPTGTGESKCPLMVKVLDAVRGSPAINVAVHVFRKAADDTWEPFASGKTSESGELHGLTTEEEFVEGIYKVEIDTKSYWKALGISPFHEHAEVVFTANDSGPRRYTIAALLSPYSYSTTAVVTNPKE | 1 | 3, 44 |
| GPTGTGESKCPLMVKVLDAVRGSPAINVAVHVFRKAADDTWEPFASGKTSESGELHGLTTEEEFVEGIYKVEIDTKYYWKALGISPFHEHAEVVFTANDSGPRRYTIAALLSPYSYSTTAVVTNPKE | 1 | 2 |
| GQGGGTHNQWNKPSKPKTNMKHMAGAAAAGAVVGGLGGYMLGSAMSRPMMHFGNDWEDRYYRENMNRYPNQVYYRPVDQYNNQNNFVHDCVNITIKQHTVTTTTKGENFTETDIKIMERVVEQMCTTQYQKESQAYYDGRRS | 1 | 3 |
| GQPHGGGWGQPHGGGWGQPHGGGWGQPH | 1 | 45 |
| GQVEVSKE | 0 | 13 |
| GQVIWVN | 1 | 10 |
| GSAMSRPMMHFGND | 1 | 19, 20 |
| GSIAAATGFV | 1 | 12 |
| GSKKRPKPGGWNTGGSRYPGQGSPGGNRYPPQGGGGWGQPHGGGWGQPHGGGWGQPHGGGWGQPHGGGWGQGGGTHSQWNKPSKPKTNMKHMAGAAAAGAVVGGLGGYMLGSAMSRPIIHFGSDYEDRYYRENMHRYPNQVYYRPMDEYSNQNNFVHDCVNITIKQHTVTTTTKGENFTETDVKMMERVVEQMCITQYERESQAYYQRGS | 1 | 2 |
| GSMSAEGYQYRALYDYKKEREEDIDLHLGDILTVNKGSLVALGFSDGQEAKPEEIGWLNGYNETTGERGDFPGTYVEYIGRKKISP | 1 | 2 |
| GSNKGAIIGLM | 1 | 5, 46, 47 |
| GSPSSRIDRTNFSNEKT | 0 | 13, 48 |
| GTAMRILGGVI | 1 | 49 |
| GTDFKYKGKL | 0 | 13 |
| GTGNTEKMAELIAKGIIESGKDY | 0 | 13 |
| GTVSFVTSPHQGAGMVGKVTVN | 1 | 7, 13 |
| GTYSFYT | 1 | 7, 13 |
| GVGKSALTIQLIQNHFVY | 1 | 7, 13 |
| GVVAIGC | 1 | 10 |
| GVVGWVKNTSKGTVTGQVQG | 1 | 7, 13, 48 |
| GWEIPEPYVWDESFRVFY | 0 | 13 |
| GYANNFLFKQG | 1 | 7, 13 |
| GYEVHHQKLVFFAEDVGSN | 1 | 13 |
| GYLTVAAVFR | 1 | 6, 7 |
| GYMLGSAMSRPIIHFGSDYED | 1 | 12 |
| HDSGYEVHHQKLVFFAEDVGSNKGA | 1 | 13 |
| HHQKLVFFAED | 1 | 13 |
| HIRLSFN | 1 | 12 |
| HLKRLLIVVVVVVLIVVVIVGALLMG | 1 | 12 |
| HMAGAAAAGAVVGGLGGYMLGSAMSRPIIHFGSDYEDRYY | 1 | 45 |
| HPETLEKFDRFKHLK | 0 | 13 |
| HQKLVFFAE | 0 | 13 |
| HSHRDFQPVLHLVALNSPLSGGMRGIRGADFQCFQQARAVGLAGTFRAFLSSRLQDLYSIVRRADRAAVPIVNLKDELLFPSWEALFSGSEGPLKPGARIFSFDGKDVLRHPTWPQKSVWHGSDPNGRRLTESYCETWRTEAPSATGQASSLLGGRLLGQSAASCHHAYIVLCIENSFMTASK | 1 | 39 |
| IIHFGSD | 1 | 12 |
| IKYLEFISDAIIHVLHSK | 1 | 3, 12 |
| IKYLEFISQAIIHVLHSR | 1 | 6, 7 |
| ILLISFLIFL | 1 | 7, 13 |
| ILPWKWPWWPWRR | 1 | 39 |
| INNTKSFEDIHQYREQIKRVKDS | 0 | 13 |
| IPAGVDAVKISM | 1 | 13 |
| IQRTPKIQVYSRHPAE | 0 | 13 |
| IQRTPKIQVYSRHPAENGKSNFLNCYVSGFHPSDIEVDLLKNGERIEKVEHSDLSFSKDWSFYLLYYTEFTPTEKDEYACRVNHVTLSQPKIVKWDRDM | 1 | 27, 50, 51 |
| ISKLEYSNFSVRY | 1 | 13, 48 |
| KCNTATCATQRLANFLVHSSNNFGAILSSTNVGSNTY | 1 | 27, 52, 53, 54 |
| KDRVQSKIGSLDNITHVPGGGN | 0 | 13 |
| KDWSFYLLYYTEFTPTEKDEYACRVNHVTLSQPKIVKWDR | 1 | 7 |
| KEGVLYVGSKTKEGVVHGVATVAEKTKEQVTNVGGAVVTGVTAVAQKTVEGAGSIAAATGFVKKDQLG | 1 | 7 |
| KELKQELFFKASATLMMS | 1 | 16 |
| KEPTKVEEPV | 0 | 10 |
| KEQVTNVGG | 1 | 12 |
| KETAAAKFERQHMDSSTSAA | 1 | 6, 7 |
| KGKKGEIKNVAD | 0 | 13 |
| KGTFEKATSEAYAYADTLKKDNGEY | 1 | 7, 13 |
| KIDAIVGRNSAKDIRTEERARVQLGNVVTAAALHGGIRISDQTTNSVETVVGKGESRVLIGNEYGGKGFWDN | 1 | 55, 56 |
| KIVFKNNA | 0 | 13 |
| KIVFKNNAGFPH | 0 | 13 |
| KKGHHEAE | 0 | 13 |
| KKLPFFAED | 0 | 13 |
| KKLVFFAED | 1 | 13 |
| KKLVFFPED | 1 | 13 |
| KKLVFPAED | 0 | 13 |
| KKLVPFAED | 0 | 13 |
| KKPVFFAED | 0 | 13 |
| KKRPKPGGWNTGGSRYPGQGSPGGNRYPPQGGGGWGQPHGGGWGQPHGGGWGQPHGGGWGQPHGGGWGQGGGTHSQWNKPSKPKTNMKHMAGAAAAGAVVGGLGGYMLGSAMSRPIIHFGSDYEDRYYRENMHRYPNQVYYRPMDEYSNQNNFVHDCVNITIKQHTVTTTTKGENFTETDVKMMERVVEQMCITQYERESQAYYQRGS | 1 | 3 |
| KLAFMLKQAELSSEKTFM | 0 | 16 |
| KLDFKDRVQSKIGSLDNITHVPGGGN | 0 | 13 |
| KLELKAASQMEFSFTMKL | 1 | 16 |
| KLVFFAE | 1 | 57 |
| KLVSSSGIKL | 0 | 10 |
| KMVLYTL | 1 | 12 |
| KNNAGFPHNV | 0 | 13 |
| KNQDKTEIPTINTIASGEPTSTPTTEAVESTVATLEDSPEVIEGPPEINTVQVTSTAV | 1 | 3 |
| KPFIARFEGRLFSRSDELKAIIKELTGE | 1 | 6, 7 |
| KPFLARFRGRIFSRSDELRTLIAAFTGE | 1 | 6, 7 |
| KPFLARVEGRIFSRSDELRAYITAYTGE | 1 | 6, 7 |
| KPFTARFEGRIFSRSDELRALITEITGE | 1 | 6, 7 |
| KPFTARISGRLFSRSDELKTIIATITGE | 1 | 6, 7 |
| KPKKTLKLVS | 0 | 10 |
| KPVDLSKVTSKCGSLGNIHHKPGGGQVEVKSEKLDF | 0 | 13 |
| KPYIARFEGRLFSRSDELRAVIEAHTGE | 1 | 6, 7 |
| KQFTLEMAFLSKALSEMK | 0 | 16 |
| KRNNFKSAEDS | 0 | 13 |
| KSAETKEPTK | 0 | 10 |
| KSSAYSLQMGATAIKQVKKLFKKWGW | 1 | 41 |
| KTEEKSELPK | 0 | 10 |
| KTLKLVSSSG | 0 | 10 |
| KTNMKHMAGAAAAGAVVGGLG | 1 | 12, 27, 58 |
| KVFERCELARTLKRLGMDGYRGISLANWMCLAKWESGYNTRATNYNAGDRSTDYGIFQINSRYWCNDGKTPGAVNACHLSCSALLQDNIADAVACAKRVVRDPQGIRAWVAWRNRCQNRDVRQYVQGCGV | 1 | 59 |
| KVFERCELARTLKRLGMDGYRGISLANWMCLAKWESGYNTRATNYNAGDRSTDYGTFQINSRYWCNDGKTPGAVNACHLSCSALLQDNIADAVACAKRVVRDPQGIRAWVAWRNRCQNRDVRQYVQGCGV | 1 | 3 |
| KVFERCELARTLKRLGMDGYRGISLANWMCLAKWESGYNTRATNYNPGDRSTDYGIFQINSRYWCNDGKTPGAVNACHLSCSALLQDNIADAVACAKRVVRDPQGIRAWVAWRNRCQNRDVRQYVQGCGV | 1 | 2 |
| KVFSKCELAHKLKAQEMDGFGGYSLANWVCMAEYESNFNTRAFNGKNANGSSDYGLFQLNNKWWCKDNKRSSSNACNIMCSKLLDENIDDDISCAKRVVRDPKGMSAWKAWVKHCKDKDLSEYLASCNL | 1 | 3 |
| KVGTKPAESD | 0 | 10 |
| KVLKAAAKAALNAVLVGANA | 1 | 1 |
| KYLLFCMENS | 1 | 12 |
| LAIEATPA | 0 | 13 |
| LANATKKVGT | 0 | 10 |
| LANWMCLAKW | 1 | 12, 13 |
| LASLIYR | 1 | 10 |
| LETFVGDQVLEIVPSNEEQIKNLLQLEAQEHLQLDFWKSPTTPGETAHVRVPFVNVQAVKVFLESQGIAYSIMIEDVQVLLDKENEEMLFNRRRERSGNFNFGAYHTLEEISQEMDNLVAEHPGLVSKVNIGSSFENRPMNVLKFSTGGDKPAIWLDAGIHAREWVTQATALWTANKIVSDYGKDPSITSILDALDIFLLPVTNPDGYVFSQTKNRMWRKTRSKVSGSLCVGVDPNRNWDAGFGGPGASSNPCSDSYHGPSANSEVEVKSIVDFIKSHGKVKAFIILHSYSQLLMFPYGYKCTKLDDFDELSEVAQKAAQSLRSLHGTKYKVGPICSVIYQASGGSIDWSYDYGIKYSFAFELRDTGRYGFLLPARQILPTAEETWLGLKAIMEHVRDHPY | 1 | 2 |
| LEVLLGSG | 0 | 13 |
| LEVLLGSGDGSLVFV | 1 | 13 |
| LKNVKSKIGSTE | 0 | 13 |
| LLGDFFRKSKEKIGKEFKRIVQRIKDFLRNLVPRTES | 1 | 31, 60 |
| LLGDLLGQTSKLVNDLTDTVGSIV | 1 | 61 |
| LLPIVGNLLKSLL | 1 | 40 |
| LMVGGVVIA | 1 | 62 |
| LPICPGGAARCQVTLRDLFDRAVVLSHYIHNLSSEMFSEFDKRYTHGRGFITKAINSCHTSSLATPEDKEQAQQMNQKDFLSLIVSILRSWNEPLYHLVTEVRGMQEAPEAILSKAVEIEEQTKRLLEGMELIVSQVHPETKENEIYPVWSGLPSLQMADEESRLSAYYNLLHCLRRDSHKIDNYLKLLKCRIIHNNNC | 1 | 2 |
| LPSGSDPAFSQPKSVLDAGLTCQGASPSSVSKPILLVPGTGTTGPQSFDSNWIPLSTQLGYTPCWISPPPFMLNDTQVNTEYMVNAITALYAGSGNNKLPVLTWSQGGLVAQWGLTFFPSIRSKVDRLMAFAPDYKGTVLAGPLDALAVSAPSVWQQTTGSALTTALRNAGGLTQIVPTTNLYSATDEIVQPQVSNSPLDSSYLFNGKNVQAQAVCGPLFVIDHAGSLTSQFSYVVGRSALRSTTGQARSADYGITDCNPLPANDLTPEQKVAAAALLAPAAAAIVAGPKQNCEPDLMPYARPFAVGKRTCSGIVTP | 1 | 2, 3 |
| LRIPCCPVNLKRLLVVVVVVVLVVVVIVGALLMGL | 1 | 3 |
| LSKVTSKCGSLGNIHHKPGGGQVE | 0 | 13 |
| LSQPKIVKWDRDM | 0 | 13 |
| LSQTFVYGGSRAKRNN | 1 | 7, 13 |
| LVEALYL | 1 | 12 |
| LVLCAASLI | 1 | 12 |
| LVLTLVVGAQAQWYRFPGEAAQGAKDMWRAYGDMKDANWKNSDKYFHARGNYDAARRGPGGRWAATVISNGREMIQGSNGRGHEDSAADQKANHWGRNGGDPNRFRPQGLPKNY | 1 | 3 |
| MAEPRQEFEVMEDHAGTYGLGDRKDQGGYTMHQDQEGDTDAGLKESPLQTPTEDGSEEPGSETSDAKSTPTAEDVTAPLVDEGAPGKQAAAQPHTEIPEGTTAEEAGIGDTPSLEDEAAGHVTQEPESGKVVQEGFLREPGPPGLSHQLMSGMPGAPLLPEGPREATRQPSGTGPEDTEGGRHAPELLKHQLLGDLHQEGPPLKGAGGKERPGSKEEVDEDRDVDESSPQDSPPSKASPAQDGRPPQTAAREATSIPGFPAEGAIPLPVDFLSKVSTEIPASEPDGPSVGRAKGQDAPLEFTFHVEITPNVQKEQAHSEEHLGRAAFPGAPGEGPEARGPSLGEDTKEADLPEPSEKQPAAAPRGKPVSRVPQLKARMVSKSKDGTGSDDKKAKTSTRSSAKTLKNRPCLSPKHPTPGSSDPLIQPSSPAVCPEPPSSPKYVSSVTSRTGSSGAKEMKLKGADGKTKIATPRGAAPPGQKGQANATRIPAKTPPAPKTPPSSGEPPKSGDRSGYSSPGSPGTPGSRSRTPSLPTPPTREPKKVAVVRTPPKSPSSAKSRLQTAPVPMPDLKNVKSKIGSTENLKHQPGGGKVQIINKKLDLSNVQSKCGSKDNIKHVPGGGSVQIVYKPVDLSKVTSKCGSLGNIHHKPGGGQVEVKSEKLDFKDRVQSKIGSLDNITHVPGGGNKKIETHKLTFRENAKAKTDHGAEIVYKSPVVSGDTSPRHLSNVSSTGSIDMVDSPQLATLADEVSASLAKQGL | 1 | 3 |
| MAGPLRAPLLLLAILAVALAVSPAAGSSPGKPPRLVGGPMDASVEEEGVRRALDFAVGEYNKASNDMYHSRALQVVRARKQIVAGVNYFLDVELGRTTCTKTQPNLDNCPFHDQPHLKRKAFCSFQIYAVPWQGTMTLSKSTCQDA | 1 | 3 |
| MAKNTSCGVQLRIRGKVQGVGFRPFVWQLAQQLNLHGDVCNDGDGVEVRLREDPEVFLVQLYQHCPPLARIDSVEREPFIWSALPTEFTIR | 1 | 3 |
| MALEKSLVRLLLLVLILLVLGWVQPSLGKESRAKKFQRQHMDSDSSPSSSSTYCNQMMRRRNMTQGRCKPVNTFVHEPLVDVQNVCFQEKVTCKNGQGNCYKSNSSMHITDCRLTNGSRYPNCAYRTSPKERHIIVACEGSPYVPVHFDASVEDST | 1 | 3 |
| MALWTRLAPLLALLALWAPAPARAFVNQHLCGSHLVEALYLVCGERGFFYTPKARREVEGPQVGALELAGGPGAGGLEGPPQKRGIVEQCCASVCSLYQLENYCN | 1 | 3 |
| MANLGCWMLVLFVATWSDLGLCKKRPKPGGWNTGGSRYPGQGSPGGNRYPPQGGGGWGQPHGGGWGQPHGGGWGQPHGGGWGQPHGGGWGQGGGTHSQWNKPSKPKTNMKHMAGAAAAGAVVGGLGGYMLGSAMSRPIIHFGSDYEDRYYRENMHRYPNQVYYRPMDEYSNQNNFVHDCVNITIKQHTVTTTTKGENFTETDVKMMERVVEQMCITQYERESQAYYQRGSSMVLFSSPPVILLISFLIFLIVG | 1 | 3 |
| MANLGCWMLVLFVATWSDLGLCKKRPKPGGWNTGGSRYPGQGSPGGNRYPPQGGGGWGQPHGGGWGQPHGGGWGQPHGGGWGQPHGGGWGQGGGTHSQWNKPSKPKTNMKHMAGAAAAGAVVGGLGGYMLGSAMSRPIIHFGSDYEDRYYRENMHRYPNQVYYRPMDQYSNQNNFVHDCVNITIKQHTVTTTTKGENFTETDVKMMERVVEQMCITQYERESQAYYQRGSSMVLFSSPPVILLISFLIFLIVG | 1 | 3 |
| MANLGYWMLVLFVATWSDLGLCKKRPKPGGWNTGGSRYPGQGSPGGNRYPPQGGGWGQPHGGGWGQPHGGGWGQPHGGGWGQPHGGGWGQAGGTHNQWNKPSKPKTNMKHMAGAAAAGAVVGGLGGYMLGSAMSRPLIHFGNDYEDRYYRENMYRYPNQVYYRPVDQYNNQNNFVHDCVNITIKQHTVTTTTKGENLTETDVKMMERVVEQMCITQYERESQAYYQRGSSMVLFSSPPVILLISFLIFLIVG | 1 | 3 |
| MAPHRPAPALLCALSLALCALSLPVRAATASRGASQAGAPQGRVPEARPNSMVVEHPEFLKAGKEPGLQIWRVEKFDLVPVPTNLYGDFFTGDAYVILKTVQLRNGNLQYDLHYWLGNECSQDESGAAAIFTVQLDDYLNGRAVQHREVQGFESATFLGYFKSGLKYKKGGVASGFKHVVPNEVVVQRLFQVKGRRVVRATEVPVSWESFNNGDCFILDLGNNIHQWCGSNSNRYERLKATQVSKGIRDNERSGRARVHVSEEGTEPEAMLQVLGPKPALPAGTEDTAKEDAANRKLAKLYKVSNGAGTMSVSLVADENPFAQGALKSEDCFILDHGKDGKIFVWKGKQANTEERKAALKTASDFITKMDYPKQTQVSVLPEGGETPLFKQFFKNWRDPDQTDGLGLSYLSSHIANVERVPFDAATLHTSTAMAAQHGMDDDGTGQKQIWRIEGSNKVPVDPATYGQFYGGDSYIILYNYRHGGRQGQIIYNWQGAQSTQDEVAASAILTAQLDEELGGTPVQSRVVQGKEPAHLMSLFGGKPMIIYKGGTSREGGQTAPASTRLFQVRANSAGATRAVEVLPKAGALNSNDAFVLKTPSAAYLWVGTGASEAEKTGAQELLRVLRAQPVQVAEGSEPDGFWEALGGKAAYRTSPRLKDKKMDAHPPRLFACSNKIGRFVIEEVPGELMQEDLATDDVMLLDTWDQVFVWVGKDSQEEEKTEALTSAKRYIETDPANRDRRTPITVVKQGFEPPSFVGWFLGWDDDYWSVDPLDRAMAELAA | 1 | 3 |
| MASHRLLLLCLAGLVFVSEAGPTGTGESKCPLMVKVLDAVRGSPAINVAVHVFRKAADDTWEPFASGKTSESGELHGLTTEEEFVEGIYKVEIDTKSYWKALGISPFHEHAEVVFTANDSGPRRYTIAALLSPYSYSTTAVVTNPKE | 1 | 3 |
| MATLEKLMKAFESLKSFQ | 0 | 16 |
| MDDDGTGQKQIWRIEGSNKVPVDPATYGQFYGGDSYIILYNYRHGGRQGQIIYNWQGAQSTQDEVAASAILTAQLDEELGGTPVQSRVVQGKEPAHLMSLFGGKPMIIYKGGTSREGGQTAPASTRLFQVRANSAGATRAVEVLPKAGALNSNDAFVLKTPSAAYLWVGTGASEAEKTGAQELLRVLRAQPVQVAEGSEPDGFWEALGGKAAYRTSPRLKDKKMDAHPPRLFACSNKIGRFVIEEVPGELMQEDLATDDVMLLDTWDQVFVWVGKDSQEEEKTEALTSAKRYIETDPANRDRRTPITVVKQGFEPPSFVGWFLGWDDDYWS | 1 | 2 |
| MDGLELLKTIRADSAY | 0 | 13 |
| MDKLLLWMFVFTSLLSEAFCQTDLKRKVFVFPRESETDHVKLIPHLEKPLQNFTLCFRTYSDLSRSQSLFSYSVKGRDNELLIYKEKVGEYSLYIGQSKVTVRGMEEYLSPVHLCTTWESSSGIVEFWVNGKPWVKKSLQREYTVKAPPSIVLGQEQDNYGGGFQRSQSFVGEFSDLYMWDYVLTPQDILFVYRDSPVNPNILNWQALNYEINGYVVIRPRVWD | 1 | 3 |
| MDVFMKGLSKAKEGVVAAAEKTKQGVAEAAGKTKEGVLYVGSKTKEGVVHGVATVAEKTKEQVTNVGGAVVTGVTAVAQKTVEGAGSIAAATGFVKKDQLGKEGYQDYEPEA | 1 | 3 |
| MDVFMKGLSKAKEGVVAAAEKTKQGVAEAAGKTKEGVLYVGSKTKEGVVHGVATVAEKTKEQVTNVGGAVVTGVTAVAQKTVEGAGSIAAATGFVKKDQLGKNEEGAPQEGILEDMPVDPDNEAYEMPSEEGYQDYEPEA | 1 | 3, 27 |
| MDVFMKGLSKAKEGVVAAAEKTKQGVAEAAGKTKEGVLYVGSKTKEGVVHGVTTVAEKTKEQVTNVGGAVVTGVTAVAQKTVEGAGNIAAATGFVKKDQMGKGEEGYPQEGILEDMPVDPSSEAYEMPSEEGYQDYEPEA | 1 | 3 |
| MDVGSKEVLMESPPDYSAAPRGRFGIPCCPVHLKRLLIVVVVVVLIVVVIVGALLMGLHMSQKHTEMVLEMSIGAPEAQQRLALSEHLVTTATFSIGSTGLVVYDYQQLLIAYKPAPGTCCYIMKIAPESIPSLEALTRKVHNFQMECSLQAKPAVPTSKLGQAEGRDAGSAPSGGDPAFLGMAVSTLCGEVPLYYI | 1 | 3 |
| MEEPQSDPSVEPPLSQETFSDLWKLLPENNVLSPLPSQAMDDLMLSPDDIEQWFTEDPGPDEAPRMPEAAPPVAPAPAAPTPAAPAPAPSWPLSSSVPSQKTYQGSYGFRLGFLHSGTAKSVTCTYSPALNKMFCQLAKTCPVQLWVDSTPPPGTRVRAMAIYKQSQHMTEVVRRCPHHERCSDSDGLAPPQHLIRVEGNLRVEYLDDRNTFRHSVVVPYEPPEVGSDCTTIHYNYMCNSSCMGGMNRRPILTIITLEDSSGNLLGRNSFEVRVCACPGRDRRTEEENLRKKGEPHHELPPGSTKRALPNNTSSSPQPKKKPLDGEYFTLQIRGRERFEMFRELNEALELKDAQAGKEPGGSRAHSSHLKSKKGQSTSRHKKLMFKTEGPDSD | 1 | 3 |
| MFSKMAKSLFLLAEKTQE | 0 | 16 |
| MGILKLQVFLIVLSVALNHLKATPIESHQVEKRKCNTATCATQRLANFLVHSSNNFGAILSSTNVGSNTYGKRNAVEVLKREPLNYLPL | 1 | 3 |
| MGTRLLPALFLVLLVLGFEVQGTQQPQQDEMPSPTFLTQVKESLSSYWESAKTAAQNLYEKTYLPAVDEKLRDLYSKSTAAMSTYTGIFTDQVLSVLKGEE | 1 | 3 |
| MIQRTPKIQVYSRHPAENGKSNFLNCYVSGFHPSDIEVDLLKNGERIEKVEHSDLSFSKDWSFYLLYYTEFTPTEKDEYACRVNHVTLSQPKIVKWDRDM | 1 | 2 |
| MKHMAGAAAAGAVV | 1 | 6, 7 |
| MKLLTGLVFCSLVLSVSSRSFFSFLGEAFDGARDMWRAYSDMREANYIGSDKYFHARGNYDAAKRGPGGAWAAEVISNARENIQRLTGHGAEDSLADQAANKWGRSGRDPNHFRPAGLPEKY | 1 | 3 |
| MKLLTSLVFCSLLLGVCHGGFFSFIGEAFQGAGDMWRAYTDMKEAGWKDGDKYFHARGNYDAAQRGPGGVWAAEKISDARESFQEFFGRGHEDTMADQEANRHGRSGKDPNYYRPPGLPAKY | 1 | 3 |
| MKLVFLVLLFLGALGLCLAGRRRSVQWCAVSQPEATKCFQWQRNMRKVRGPPVSCIKRDSPIQCIQAIAENRADAVTLDGGFIYEAGLAPYKLRPVAAEVYGTERQPRTHYYAVAVVKKGGSFQLNELQGLKSCHTGLRRTAGWNVPIGTLRPFLNWTGPPEPIEAAVARFFSASCVPGADKGQFPNLCRLCAGTGENKCAFSSQEPYFSYSGAFKCLRDGAGDVAFIRESTVFEDLSDEAERDEYELLCPDNTRKPVDKFKDCHLARVPSHAVVARSVNGKEDAIWNLLRQAQEKFGKDKSPKFQLFGSPSGQKDLLFKDSAIGFSRVPPRIDSGLYLGSGYFTAIQNLRKSEEEVAARRARVVWCAVGEQELRKCNQWSGLSEGSVTCSSASTTEDCIALVLKGEADAMSLDGGYVYTAGKCGLVPVLAENYKSQQSSDPDPNCVDRPVEGYLAVAVVRRSDTSLTWNSVKGKKSCHTAVDRTAGWNIPMGLLFNQTGSCKFDEYFSQSCAPGSDPRSNLCALCIGDEQGENKCVPNSNERYYGYTGAFRCLAENAGDVAFVKDVTVLQNTDGNNNEAWAKDLKLADFALLCLDGKRKPVTEARSCHLAMAPNHAVVSRMDKVERLKQVLLHQQAKFGRNGSDCPDKFCLFQSETKNLLFNDNTECLARLHGKTTYEKYLGPQYVAGITNLKKCSTSPLLEACEFLRK | 1 | 3 |
| MKVIFLKDVKG | 1 | 7, 13 |
| MLEGKVKWFNSEKGFGFIEVEG | 1 | 12 |
| MLEGKVKWFNSEKGFGFIEVEGQDDVFVHFSAIQG | 1 | 12 |
| MLKLLDNWDSVTSTFSKLREQLGPVTQEFWDNLEKETEGLRQEMSKDLEEVKAKVQPYLDDFQKKWQEEMELYRQKVEPLRAELQEGARQKLHELQEKLSPLGEEMRDRARAHVDALRTHLAPYSDELRQRLAARLEALKENGGARLAEYHAKATEHLSTLSEKAKPALEDLRQGLLPVLESFKVSFLSALEEYTKKLNTQ | 1 | 2 |
| MLPGLALLLLAAWTARALEVPTDGNAGLLAEPQIAMFCGRLNMHMNVQNGKWDSDPSGTKTCIDTKEGILQYCQEVYPELQITNVVEANQPVTIQNWCKRGRKQCKTHPHFVIPYRCLVGEFVSDALLVPDKCKFLHQERMDVCETHLHWHTVAKETCSEKSTNLHDYGMLLPCGIDKFRGVEFVCCPLAEESDNVDSADAEEDDSDVWWGGADTDYADGSEDKVVEVAEEEEVAEVEEEEADDDEDDEDGDEVEEEAEEPYEEATERTTSIATTTTTTTESVEEVVREVCSEQAETGPCRAMISRWYFDVTEGKCAPFFYGGCGGNRNNFDTEEYCMAVCGSAMSQSLLKTTQEPLARDPVKLPTTAASTPDAVDKYLETPGDENEHAHFQKAKERLEAKHRERMSQVMREWEEAERQAKNLPKADKKAVIQHFQEKVESLEQEAANERQQLVETHMARVEAMLNDRRRLALENYITALQAVPPRPRHVFNMLKKYVRAEQKDRQHTLKHFEHVRMVDPKKAAQIRSQVMTHLRVIYERMNQSLSLLYNVPAVAEEIQDEVDELLQKEQNYSDDVLANMISEPRISYGNDALMPSLTETKTTVELLPVNGEFSLDDLQPWHSFGADSVPANTENEVEPVDARPAADRGLTTRPGSGLTNIKTEEISEVKMDAEFRHDSGYEVHHQKLVFFAEDVGSNKGAIIGLMVGGVVIATVIVITLVMLKKKQYTSIHHGVVEVDAAVTPEERHLSKMQQNGYENPTYKFFEQMQN | 1 | 3 |
| MLPSLALLLLAAWTVRALEVPTDGNAGLLAEPQIAMFCGKLNMHMNVQNGKWESDPSGTKTCIGTKEGILQYCQEVYPELQITNVVEANQPVTIQNWCKRGRKQCKTHTHIVIPYRCLVGEFVSDALLVPDKCKFLHQERMDVCETHLHWHTVAKETCSEKSTNLHDYGMLLPCGIDKFRGVEFVCCPLAEESDSVDSADAEEDDSDVWWGGADTDYADGGEDKVVEVAEEEEVADVEEEEADDDEDVEDGDEVEEEAEEPYEEATERTTSTATTTTTTTESVEEVVRVPTTAASTPDAVDKYLETPGDENEHAHFQKAKERLEAKHRERMSQVMREWEEAERQAKNLPKADKKAVIQHFQEKVESLEQEAANERQQLVETHMARVEAMLNDRRRLALENYITALQAVPPRPHHVFNMLKKYVRAEQKDRQHTLKHFEHVRMVDPKKAAQIRSQVMTHLRVIYERMNQSLSLLYNVPAVAEEIQDEVDELLQKEQNYSDDVLANMISEPRISYGNDALMPSLTETKTTVELLPVNGEFSLDDLQPWHPFGVDSVPANTENEVEPVDARPAADRGLTTRPGSGLTNIKTEEISEVKMDAEFGHDSGFEVRHQKLVFFAEDVGSNKGAIIGLMVGGVVIATVIVITLVMLKKKQYTSIHHGVVEVDAAVTPEERHLSKMQQNGYENPTYKFFEQMQN | 1 | 3 |
| MLPSLALLLLAAWTVRALEVPTDGNAGLLAEPQIAMFCGKLNMHMNVQNGKWESDPSGTKTCIGTKEGILQYCQEVYPELQITNVVEANQPVTIQNWCKRGRKQCKTHTHIVIPYRCLVGEFVSDALLVPDKCKFLHQERMDVCETHLHWHTVAKETCSEKSTNLHDYGMLLPCGIDKFRGVEFVCCPLAEESDSVDSADAEEDDSDVWWGGADTDYADGGEDKVVEVAEEEEVADVEEEEADDDEDVEDGDEVEEEAEEPYEEATERTTSTATTTTTTTESVEEVVRVPTTAASTPDAADKYLETPGDENEHAHFQKAKERLEAKHRERMSQVMREWEEAERQAKNLPKADKKAVIQHFQEKVESLEQEAANERQQLVETHMARVEAMLNDRRRLALENYITALQAVPPRPHHVFNMLKKYVRAEQKDRQHTLKHFEHVRMVDPKKAAQIRSQVMTHLRVIYERMNQSLSLLYNVPAVAEEIQDEVDELLQKEQNYSDDVLANMISEPRISYGNDALMPSLTETKTTVELLPVNGEFSLDDLQPWHPFGVDSVPANTENEVEPVDARPAADRGLTTRPGSGLTNIKTEEISEVKMDAEFGHDSGFEVRHQKLVFFAEDVGSNKGAIIGLMVGGVVIATVIVITLVMLKKKQYTSIHHGVVEVDAAVTPEERHLSKMQQNGYENPTYKFFEQMQN | 1 | 3 |
| MLSLKESAKMFFATKELQ | 0 | 16 |
| MLSNTTAIAEAWARL | 1 | 6, 7 |
| MLTFAEFKSMELKSQLAK | 0 | 16 |
| MLVLFVATWSDLGLCKKRPKPGGWNTGGSRYPGQGSPGGNRYPPQGGGGWGQPHGGGWGQPHGGGWGQPHGGGWGQPHGGGWGQGGGTHSQWNKPSKPKTNMKHMAGAAAGAVVGGLGGYMLGSAMSRPIIHFGSDYEDRYYRENMHRYPNQVYYRPMDEYSNQNNFVHDCVNITIKQHTVTTTTKGENFTETDVKMMERVVEQMCITQYERESQAYYQRGSSMVLFSSPPVILLISFLIFLIVG | 1 | 3 |
| MMNNNGNQVSNLSNALRQVNIGNRNSNTTTDQSNINFEFSTGVNNNNNNNSSSNNNNVQNNNSGRNGSQNNDNENNIKNTLEQHRQQQQ | 1 | 12 |
| MMNNNGNQVSNLSNALRQVNIGNRNSNTTTDQSNINFEFSTGVNNNNNNNSSSNNNNVQNNNSGRNGSQNNDNENNIKNTLEQHRQQQQAFSDMSHV | 1 | 3 |
| MPEEELL | 0 | 13 |
| MPEEELLNAPGETYVVTL | 1 | 7, 13 |
| MPRPRLLAALCGALLCAPSLLVALDICSKNPCHNGGLCEEISQEVRGDVFPSYTCTCLKGYAGNHCETKCVEPLGMENGNIANSQIAASSVRVTFLGLQHWVPELARLNRAGMVNAWTPSSNDDNPWIQVNLLRRMWVTGVVTQGASRLASHEYLKAFKVAYSLNGHEFDFIHDVNKKHKEFVGNWNKNAVHVNLFETPVEAQYVRLYPTSCHTACTLRFELLGCELNGCANPLGLKNNSIPDKQITASSSYKTWGLHLFSWNPSYARLDKQGNFNAWVAGSYGNDQWLQVDLGSSKEVTGIITQGARNFGSVQFVASYKVAYSNDSANWTEYQDPRTGSSKIFPGNWDNHSHKKNLFETPILARYVRILPVAWHNRIALRLELLGC | 1 | 3 |
| MPYVFSFKMPQEQGQMFQYYPVYMVLPWEQPQQTVPRSPQQTRQQQ | 1 | 12 |
| MQTLSERLKKRRIALKY | 0 | 13 |
| MQYKVILNGKTLKGETTTEAVDAATFEKVVKQFFNDNGVDGEWTYDDATKTFTVTE | 1 | 3 |
| MRCISRLPAVLLILSVALGHLRATPVGSGTNPQVDKRKCNTATCATQRLANFLVRSSNNLGPVLPPTNVGSNTYGKRNVAEDPNRESLDFLLL | 1 | 3 |
| MRGSHHHHHHGSNANPNANPNANPNANPNANPNANPNANPNANPNANPNANPNANPNANPNANPNANPNANPNANPNANPNANPNANPRSMDAEFRHDSGYEVHHQKLVFFAEDVGSNKGAIIGLMVGGVVIA | 1 | 3 |
| MSRSVALAVLALLSLSGLEAIQRTPKIQVYSRHPAENGKSNFLNCYVSGFHPSDIEVDLLKNGERIEKVEHSDLSFSKDWSFYLLYYTEFTPTEKDEYACRVNHVTLSQPKIVKWDRDM | 1 | 3 |
| MSSYAFFVQTCREEHK | 1 | 12 |
| MSTAQSLKSVDYEVFGRVQGVCFRMYTEDEARKIGVVGWVKNTSKGTVTGQVQGPEDKVNSMKSWLSKVGSPSSRIDRTNFSNEKTISKLEYSNFSIRY | 1 | 3 |
| MSTYTGIFTDQ | 1 | 12 |
| MVGKVTVN | 0 | 13 |
| MVKSHIGSWILVLFVAMWSDVGLCKKRPKPGGGWNTGGSRYPGQGSPGGNRYPPQGGGGWGQPHGGGWGQPHGGGWGQPHGGGWGQPHGGGGWGQGGTHSQWNKPSKPKTNMKHVAGAAAAGAVVGGLGGYMLGSAMSRPLIHFGNDYEDRYYRENMYRYPNQVYYRPVDQYNNQNTFVHDCVNITVKQHTVTTTTKGENFTETDIKMMERVVEQMCITQYQRESQAYYQRGASVILFSSPPVILLISFLIFLIVG | 1 | 3 |
| MVKVTFNSALAQKEAKKDEPKSGEEALIIPPDAVAVDCKDPDDVVPVGQRRAWCWCMCFGLAFMLAGVILGGAYLYKYFALQPDDVYYCGIKYIKDDVILNEPSADAPAALYQTIEENIKIFEEEEVEFISVPVPEFADSDPANIVHDFNKKLTAYLDLNLDKCYVIPLNTSIVMPPRNLLELLINIKAGTYLPQSYLIHEHMVITDRIENIDHLGFFIYRLCHDKETYKLQRRETIKGIQKREASNCFAIRHFENKFAVETLICS | 1 | 3 |
| NAGDVAFV | 1 | 6, 7 |
| NAGSVQAVA | 1 | 36 |
| NAKAGLSQT | 0 | 13 |
| NAPGETY | 0 | 13 |
| NDFQKQQKQA | 1 | 10 |
| NEQLAKQKGCMACHDLKAKKVGPAYADVAKKYAGRKDAVDYLAGKIKKGGSGVWGSVPMPPQNVTDAEAKQLAQWILSIK | 1 | 2 |
| NFAAILSS | 1 | 6, 7, 11 |
| NFGAALSS | 1 | 6, 7, 11 |
| NFGAIASS | 1 | 6, 7, 11 |
| NFGAILSS | 1 | 6, 7, 11, 17, 63 |
| NFGSVQFV | 1 | 6, 7, 17, 36, 64 |
| NFGSVQFVA | 1 | 12 |
| NFLNCYVSGFH | 1 | 12 |
| NFLVHSS | 1 | 12 |
| NFLVHSSNN | 1 | 6, 7 |
| NFLVHSSNNFGAILSS | 1 | 7 |
| NFNYNNNLQG | 1 | 10 |
| NGERIEKVEHSDLSFSKD | 0 | 13 |
| NGKSNFLNCYVSG | 0 | 13 |
| NHVTLSQ | 1 | 6, 7 |
| NLKHQPGGGKVQIVYKEVD | 1 | 7, 13 |
| NLKHQPGGGKVQIVYKPVDLSKVTSKCGSLGNIHHKPGGGQVE | 1 | 7, 13 |
| NLQGYQAGFQ | 1 | 10 |
| NLSLFDQ | 1 | 10 |
| NNFGAILSST | 1 | 12, 52 |
| PEDKVNSMKSWLSKV | 0 | 13, 48 |
| PFTAATLEEKLNKIFEKLGMY | 1 | 7, 13 |
| PGGGKNAEVYKPV | 0 | 13 |
| PGGGKVQIVEKPV | 0 | 13 |
| PGGGKVQIVYKPV | 1 | 13 |
| PGGGKVYKPV | 0 | 13 |
| PHNVVFDEDDEIP | 0 | 13 |
| PKIKAFLA | 0 | 13 |
| PLLSAYVARLSA | 0 | 13 |
| PQGGRGNYKN | 1 | 10 |
| PQGGYQQYN | 1 | 6, 7 |
| PSKARIIRY | 0 | 13 |
| PTGKDEYACRVNHVT | 0 | 13 |
| PVQTEEKTEE | 0 | 10 |
| QGGYQQYNPQ | 0 | 10 |
| QGVSFRMYTEDEARKI | 0 | 48 |
| QISFADYNLLDLLRIHQVLN | 1 | 7, 13 |
| QKGGSKGRLPSEFSQFPHGQKGQHYSGQKGKQQTESKGSFSIQYTYHVDANDHDQSRKSQQYDLNALHKTTKSQRHLGGSQQLLHNKQEGRDHDKSKGHFHRVVIHHKGGKAHRGTQNPSQDQGNSPSGKGISSQYSNTEER | 1 | 12 |
| QKLVFFA | 0 | 13 |
| QKLVFFAEDVGSNKGAIIGLMVGGVVIA | 1 | 7 |
| QKPKLLYCSNGGYFLRIFPDGKVDGTRDRSDPYIQLQFYAESVGEVYIKSLETGQYLAMDSDGQLYASQSPSEECLFLERLEENNYNTYKSKVHADKDWFVGIKKNGKTKPGSRTHFGQKAILFLPLPVSSD | 1 | 3 |
| QKTVEGAGSIAAATGEV | 0 | 2 |
| QNKELCDVTSSTGLLDSIKVMASHVKEQLKDKGTSEVAQPIVSPDPTDCADILLNGYRSSGGYRIWPKSWMTVGTLNVYCDMETDGGGWTVIQRRGNYGNPSDYFYKPWKNYKLGFGNIEKDFWLGNDRIFALTNQRNYMIRFDLKDKENDTRYAIYQDFWIENEDYLYCLHIGNYSGDAGNSFGRHNGHNFSTIDKDHDTHETHCAQTYKGGWWYDRCHESNLNGLYLNGEHNSYADGIEWRAWKGYHYSLPQVEMKIRPVEFNIIGN | 1 | 2 |
| QQEVINK | 1 | 10 |
| QQQYNPQGGY | 0 | 10 |
| QRLANFLVH | 1 | 6, 7 |
| QTAPVPMPD | 0 | 13 |
| QTAPVPMPDLKNVKSKIGSTE | 0 | 13 |
| QTAPVPMPDLKNVKSKIGSTENLKHQPGGGKVQIVY | 0 | 13 |
| RCELARTLKR | 1 | 12, 13 |
| RFRAVTSAYYRGAVG | 0 | 13 |
| RHFWQQDEPPQSPWDRVKDLATVYVDVLKDSGRDYVSQFEGSALGKQLNLKLLDNWDSVTSTFSKLREQLGPVTQEFWDNLEKETEGLRQEMS | 1 | 7 |
| RLDKQGNFNAWVAGSYGNDQWLQVDLGSSKEVTGIITQGARNFGSVQFVA | 1 | 32, 36 |
| RPDFSLEPPYTGPSK | 0 | 13 |
| RPPQFTRAQWFAIQHISLNPPRCTIAMRAINNYRWRCKNQNTFLRTTFANVVNVCGNQSIRCPHNRTLNNCHRSRFRVPLLHCDLINPGAQNISNCTYADRPGRRFYVVACDNRDPRDSPRYPVVPVHLDTTI | 1 | 65 |
| RQGNINIVA | 1 | 10 |
| RQGVEDAFYTLVREIRQHK | 1 | 7, 13 |
| RRRSVQWCTVSQPEATKCFQWQRNMRRVRGPPVSCIKRDSPIQCIQAIAENRADAVTLDGGFIYEAGLAPYKLRPVAAEVYGTERQPRTHYYAVAVVKKGGSFQLNELQGLKSCHTGLRRTAGWNVPIGTLRPFLNWTGPPEPIEAAVARFFSASCVPGADKGQFPNLCRLCAGTGENKCAFSSQEPYFSYSGAFKCLRDGAGDVAFIRESTVFEDLSDEAERDEYELLCPDNTRKPVDKFKDCHLARVPSHAVVARSVNGKEDAIWNLLRQAQEKFGKDKSPKFQLFGSPSGQKDLLFKDSAIGFSRVPPRIDSGLYLGSGYFTAIQNLRKSEEEVAARRARVVWCAVGEQELRKCNQWSGLSEGSVTCSSASTTEDCIALVLKGEADAMSLDGGYVYTAGKCGLVPVLAENYKSQQSSDPDPNCVDRPVEGYLAVAVVRRSDTSLTWNSVKGKKSCHTAVDRTAGWNIPMGLLFNQTGSCKFDEYFSQSCAPGSDPRSNLCALCIGDEQGENKCVPNSNERYYGYTGAFRCLAENAGDVAFVKDVTVLQNTDGNNNDAWAKDLKLADFALLCLDGKRKPVTEARSCHLAMAPNHAVVSRMDKVERLKQVLLHQQAKFGRNGSDCPDKFCLFQSETKNLLFNDNTECLARLHGKTTYEKYLGPQYVAGITNLKKCSTSPLLEACEFLRK | 1 | 2 |
| RSFFSFLGEAFD | 1 | 12 |
| RSWFSFLGEAY | 1 | 7 |
| RVEKVAILGLMVLA | 1 | 6, 7 |
| RVQGVCFRMYTEDEAR | 1 | 12 |
| RYQGYQAYNA | 1 | 10 |
| RYTHGRGFITKAINS | 1 | 12 |
| SAFMEKMLLLEKQFKAST | 1 | 16 |
| SAMSRPIIHFGSDYEDRYYRENMHRYPN | 1 | 7 |
| SAPNLATLVKVTTNHFTHEEAMMD | 1 | 7, 13 |
| SDIEVDLLK | 1 | 12 |
| SEDLKKHGVTVLTALGAILK | 0 | 13 |
| SEFSVPSGEK | 0 | 13 |
| SELNIYQY | 1 | 10 |
| SETSRTAFGGRRAVPPNNSNAAEDDLPTVELQGVVPRGVNLQEFLNVTSVHLFKERWDTNKVDHHTDKYENNKLIVRRGQSFYVQIDFSRPYDPRRDLFRVEYVIGRYPQENKGTYIPVPIVSELQSGKWGAKIVMREDRSVRLSIQSSPKCIVGKFRMYVAVWTPYGVLRTSRNPETDTYILFNPWCEDDAVYLDNEKEREEYVLNDIGVIFYGEVNDIKTRSWSYGQFEDGILDTCLYVMDRAQMDLSGRGNPIKVSRVGSAMVNAKDDEGVLVGSWDNIYAYGVPPSAWTGSVDILLEYRSSENPVRYGQCWVFAGVFNTFLRCLGIPARIVTNYFSAHDNDANLQMDIFLEEDGNVNSKLTKDSVWNYHCWNEAWMTRPDLPVGFGGWQAVDSTPQENSDGMYRCGPASVQAIKHGHVCFQFDAPFVFAEVNSDLIYITAKKDGTHVVENVDATHIGKLIVTKQIGGDGMMDITDTYKFQEGQEEERLALETALMYGAKKPLNTEGVMKSRSNVDMDFEVENAVLGKDFKLSITFRNNSHNRYTITAYLSANITFYTGVPKAEFKKETFDVTLEPLSFKKEAVLIQAGEYMGQLLEQASLHFFVTARINETRDVLAKQKSTVLTIPEIIIKVRGTQVVGSDMTVTVEFTNPLKETLRNVWVHLDGPGVTRPMKKMFREIRPNSTVQWEEVCRPWVSGHRKLIASMSSDSLRHVYGELDVQIQRRPSM | 1 | 2, 3 |
| SFFSFLGEAFD | 1 | 3, 6, 7, 32 |
| SFNNGDCFILD | 1 | 6, 7 |
| SGYEVHHQKLVFFAEDVGSNK | 1 | 13 |
| SKLEYSNFSIRY | 1 | 12 |
| SLACQCLVRTP | 1 | 12 |
| SLLNIVV | 1 | 10 |
| SLVFVPSEFS | 0 | 13 |
| SMVLFSSPPV | 1 | 7, 13 |
| SMVLFSSPPVILLISFLIFLIVG | 1 | 12 |
| SNFLNCYVSGFHPSDIEVDLL | 1 | 7 |
| SNFLNCYVSGFHPSDIEVDLLK | 1 | 6, 7, 66 |
| SNLSNALRQVNIGNRNSNTTTDQSNINFEF | 1 | 12 |
| SNNFGAIL | 1 | 6, 7 |
| SNNFGAILSS | 1 | 17, 67 |
| SRPMMHFG | 1 | 19, 20 |
| SRPMMHFGNDWEDRY | 1 | 19, 20 |
| SSPGKPPRLVGGPMDASVEEEGVRRALDFAVGEYNKASNDMYHSRALQVVRARKQIVAGVNYFLDVELGRTTCTKTQPNLDNCPFHDQPHLKRKAFCSFQIYAVPWQGTMTLSKSTCQDA | 1 | 2 |
| SSPPVILLIS | 1 | 7, 13 |
| SSSGIKLANA | 0 | 10 |
| SSTSAASSSNY | 1 | 68 |
| STAQSLKSVDYEVFGRV | 0 | 13, 48 |
| STNVKTAFEMVILDIYNNV | 1 | 7, 13 |
| SYGGEGIGNVAVAGELPVAGKTAVAGRVPIIGAVGFGGPAGAAGAVSIAGR | 1 | 6, 7 |
| TAFTILA | 1 | 10 |
| TEAEMKA | 0 | 13 |
| TEEKTEEKSE | 0 | 10 |
| TESKEKITQYIYHVLNGEIL | 1 | 7, 13 |
| TKFSSFALLAQKEMLKME | 0 | 16 |
| TKPAESDKKE | 0 | 10 |
| TKRPRFLYEIAMALNSD | 1 | 7, 13 |
| TKVEEPVKKE | 0 | 10 |
| TLLKKVLKAAAK | 1 | 1 |
| TLLKKVLKAAAKAALNAVLVGANA | 1 | 1 |
| TLSIYQY | 1 | 10 |
| TMMKFQLLKSAEEKLFAS | 0 | 16 |
| TNVGSNTY | 1 | 6, 17, 69 |
| TPANLKALEAQKQKEQR | 0 | 13 |
| TPIESHQVEKRKCNTATCATQRLANFLVHSSNNFGAILSSTNVGSNTY | 1 | 3, 70 |
| TQQPQQDEMPSPTFLTQVKESLSSYWESAKTAAQNLYEKTYLPAVDEKLRDLYSKSTAAMSTYTGIFTDQVLSVLKGEE | 1 | 71 |
| TRRTTFESVGRWLDELKIHSD | 0 | 13 |
| TVIAFLA | 1 | 10 |
| TVNGVGEVTATAVQGVAV | 1 | 7, 13 |
| TYKLINGKTLKGETTTEA | 0 | 13 |
| VDLSKVTSK | 0 | 13 |
| VFMKGLSKAKEGVVAA | 1 | 7 |
| VGGAVVTGV | 1 | 6, 7, 13 |
| VHDCVNITIK | 1 | 12, 13 |
| VHGVATVAEKT | 1 | 12 |
| VHHPKLVFFAEDVGS | 1 | 13 |
| VHHQEKLVFFAEDPGS | 1 | 13 |
| VHHQEKLVFFAEDVPS | 1 | 13 |
| VHHQEKLVFFAEPVGS | 1 | 13 |
| VHHQEKLVFFAPDVGS | 0 | 13 |
| VHHQKLVFFAEDV | 1 | 3, 13 |
| VHHQKLVFFAEDVGS | 1 | 13 |
| VHHQKLVPFAEDVGS | 0 | 13 |
| VHHQPLVFFAEDVGS | 1 | 13 |
| VHPQKLVFFAEDVGS | 1 | 13 |
| VHSSNNFGAILSS | 1 | 72 |
| VKKEEKPVQT | 0 | 10 |
| VKSEKLDFKDRVQSKIGSLDNITHVPGGGN | 0 | 13 |
| VLSEGEWQLVLHVWAKVEA | 1 | 7, 13 |
| VNGMELSKQILQENPH | 0 | 13 |
| VPHQKLVFFAEDVGS | 1 | 7, 13 |
| VPSNEEQIKKLLELEAKKHLQY | 0 | 13 |
| VPSNEEQIKNLLQLEAQEHLQY | 0 | 13 |
| VQQNYQA | 1 | 10 |
| VTGIITQGAR | 1 | 36 |
| VTGVTAVAQKTV | 1 | 2 |
| VTGVTAVQKTV | 1 | 6, 7 |
| VTIKANLIFANGFTQTAEFKG | 1 | 7, 13 |
| VTLDTKGTY | 0 | 13 |
| VTNVGGAVVTGVTAVA | 1 | 2, 7, 13 |
| VTSKCGSLGNIHHKPGGG | 0 | 13 |
| VTVKVNAVKVTV | 1 | 6, 7 |
| WVAGSYGNDQWLQVDLGSSKEVTGIITQGARNFGSVQFVA | 1 | 36 |
| YDYQEKSPREVTMKKGD | 0 | 13 |
| YEAGKEKYVKELPEHLKPFETLLSQ | 0 | 13 |
| YEVHHQKLVFFAEDVGS | 1 | 13 |
| YEVHHQKLVFFAEDVGSNKGAIIGLM | 1 | 19, 26 |
| YGNLISL | 1 | 10 |
| YKMTQTELATKAGVK | 0 | 13 |
| YKQQSIQLIEAGVTKR | 0 | 13 |
| YNNNLQGYQA | 1 | 10 |
| YNPQGGYQQQ | 0 | 10 |
| YNPQGGYQQY | 0 | 10 |
| YQNYQGYSGY | 1 | 10 |
| YQQGGYQQYN | 1 | 10 |
| YQQYNPQGGY | 0 | 10 |
| YSGYQQGGYQ | 1 | 10 |
| YSQNGNQQQG | 1 | 10 |
| YTIAALLSPYS | 1 | 3, 6, 7, 32, 73 |
| YTSPHQGAGMV | 0 | 13 |

**Bibliography**

1. R. Feder, A. Dagan, and A. Mor, “Structure-activity relationship study of antimicrobial dermaseptin S4 showing the consequences of peptide oligomerization on selective cytotoxicity.,” J. Biol. Chem., vol. 275, no. 6, pp. 4230–8, Feb. 2000.
2. S. Yoon and W. J. Welsh, “Detecting hidden sequence propensity for amyloid fibril formation.,” Protein Sci., vol. 13, no. 8, pp. 2149–60, Aug. 2004.
3. O. Conchillo-Solé, N. S. de Groot, F. X. Avilés, J. Vendrell, X. Daura, and S. Ventura, “AGGRESCAN: a server for the prediction and evaluation of ‘hot spots’ of aggregation in polypeptides.,” BMC Bioinformatics, vol. 8, no. 65, pp. 1–17, Jan. 2007.
4. K. F. DuBay, A. P. Pawar, F. Chiti, J. Zurdo, C. M. Dobson, and M. Vendruscolo, “Prediction of the absolute aggregation rates of amyloidogenic polypeptide chains.,” J. Mol. Biol., vol. 341, no. 5, pp. 1317–26, Aug. 2004.
5. M. G. Cottingham, J. L. A. Voskuil, and D. J. T. Vaux, “The intact human acetylcholinesterase C-terminal oligomerization domain is alpha-helical in situ and in isolation, but a shorter fragment forms beta-sheet-rich amyloid fibrils and protofibrillar oligomers.,” Biochemistry, vol. 42, no. 36, pp. 10863–73, Sep. 2003.
6. M. J. Thompson, S. a Sievers, J. Karanicolas, M. I. Ivanova, D. Baker, and D. Eisenberg, “The 3D profile method for identifying fibril-forming segments of proteins.,” in Proceedings of the National Academy of Sciences of the United States of America, 2006, vol. 103, no. 11, pp. 4074–4078.
7. J. Tian, N. Wu, J. Guo, and Y. Fan, “Prediction of amyloid fibril-forming segments based on a support vector machine.,” BMC Bioinformatics, vol. 10 Suppl 1, no. S45, pp. 1–8, Jan. 2009.
8. M. J. Bayro, T. Maly, N. R. Birkett, C. E. Macphee, C. M. Dobson, and R. G. Griffin, “High-resolution MAS NMR analysis of PI3-SH3 amyloid fibrils: backbone conformation and implications for protofilament assembly and structure .,” Biochemistry, vol. 49, no. 35, pp. 7474–7484, Sep. 2010.
9. Y. Singh, P. C. Sharpe, H. N. Hoang, A. J. Lucke, A. W. McDowall, S. P. Bottomley, and D. P. Fairlie, “Amyloid formation from an α-helix peptide bundle is seeded by 3(10)-helix aggregates.,” Chemistry, vol. 17, no. 1, pp. 151–160, Jan. 2011.
10. S. Maurer-Stroh, M. Debulpaep, N. Kuemmerer, M. Lopez de la Paz, I. C. Martins, J. Reumers, K. L. Morris, A. Copland, L. C. Serpell, L. Serrano, J. W. H. Schymkowitz, and F. Rousseau, “Exploring the sequence determinants of amyloid structure using position-specific scoring matrices.,” Nat. Methods, vol. 7, no. 3, pp. 237–242, Mar. 2010.
11. R. Azriel and E. Gazit, “Analysis of the minimal amyloid-forming fragment of the islet amyloid polypeptide. An experimental support for the key role of the phenylalanine residue in amyloid formation.,” J. Biol. Chem., vol. 276, no. 36, pp. 34156–61, Sep. 2001.
    1. C. Tsolis, N. C. Papandreou, V. A. Iconomidou, and S. J. Hamodrakas, “A consensus method for the prediction of ‘aggregation-prone’ peptides in globular proteins.,” PLoS One, vol. 8, no. 1, p. e54175, Jan. 2013.
12. A.-M. Fernandez-Escamilla, F. Rousseau, J. W. H. Schymkowitz, and L. Serrano, “Prediction of sequence-dependent and mutational effects on the aggregation of peptides and proteins.,” Nat. Biotechnol., vol. 22, no. 10, pp. 1302–1306, Oct. 2004.
13. J. K. Ghosh, D. Shaool, P. Guillaud, L. Cicéron, D. Mazier, I. Kustanovich, Y. Shai, and A. Mor, “Selective cytotoxicity of dermaseptin S3 toward intraerythrocytic Plasmodium falciparum and the underlying molecular basis.,” J. Biol. Chem., vol. 272, no. 50, pp. 31609–16, Dec. 1997.
14. C. Auvynet, C. El Amri, C. Lacombe, F. Bruston, J. Bourdais, P. Nicolas, and Y. Rosenstein, “Structural requirements for antimicrobial versus chemoattractant activities for dermaseptin S9.,” FEBS J., vol. 275, no. 16, pp. 4134–51, Aug. 2008.
15. M. Emily, A. Talvas, and C. Delamarche, “MetAmyl: a METa-predictor for AMYLoid proteins.,” PLoS One, vol. 8, no. 11, p. e79722, Jan. 2013.
16. Y. Mazor, S. Gilead, I. Benhar, and E. Gazit, “Identification and characterization of a novel molecular-recognition and self-assembly domain within the islet amyloid polypeptide.,” J. Mol. Biol., vol. 322, no. 5, pp. 1013–1024, Oct. 2002.
17. E. T. Jaikaran, C. E. Higham, L. C. Serpell, J. Zurdo, M. Gross, A. Clark, and P. E. Fraser, “Identification of a novel human islet amyloid polypeptide beta-sheet domain and factors influencing fibrillogenesis.,” J. Mol. Biol., vol. 308, no. 3, pp. 515–525, May 2001.
18. B. Ma and R. Nussinov, “Stabilities and conformations of Alzheimer’s beta -amyloid peptide oligomers (Abeta 16-22, Abeta 16-35, and Abeta 10-35): Sequence effects.,” Proc. Natl. Acad. Sci. U. S. A., vol. 99, no. 22, pp. 14126–14131, Oct. 2002.
19. M. Gasset, M. A. Baldwin, D. H. Lloyd, J. M. Gabriel, D. M. Holtzman, F. Cohen, R. Fletterick, and S. B. Prusiner, “Predicted alpha-helical regions of the prion protein when synthesized as peptides form amyloid.,” Proc. Natl. Acad. Sci. U. S. A., vol. 89, no. 22, pp. 10940–10944, Nov. 1992.
20. M. Kamihira, A. Naito, S. Tuzi, A. Y. Nosaka, and H. Saitô, “Conformational transitions and fibrillation mechanism of human calcitonin as studied by high-resolution solid-state 13C NMR.,” Protein Sci., vol. 9, no. 5, pp. 867–877, May 2000.
21. T. Arvinte, A. Cudd, and A. F. Drake, “The structure and mechanism of formation of human calcitonin fibrils.,” J. Biol. Chem., vol. 268, no. 9, pp. 6415–6422, Mar. 1993.
22. M. Reches, Y. Porat, and E. Gazit, “Amyloid fibril formation by pentapeptide and tetrapeptide fragments of human calcitonin.,” J. Biol. Chem., vol. 277, no. 38, pp. 35475–35480, Sep. 2002.
23. C. E. MacPhee and C. M. Dobson, “Chemical dissection and reassembly of amyloid fibrils formed by a peptide fragment of transthyretin.,” J. Mol. Biol., vol. 297, no. 5, pp. 1203–1215, Apr. 2000.
24. C. P. Jaroniec, C. E. MacPhee, N. S. Astrof, C. M. Dobson, and R. G. Griffin, “Molecular conformation of a peptide fragment of transthyretin in an amyloid fibril.,” Proc. Natl. Acad. Sci. U. S. A., vol. 99, no. 26, pp. 16748–16753, Dec. 2002.
25. L. C. Serpell, “Alzheimer’s amyloid fibrils: structure and assembly.,” Biochim. Biophys. Acta, vol. 1502, no. 1, pp. 16–30, Jul. 2000.
26. M. Zhang, J. Zhao, and J. Zheng, “Molecular understanding of a potential functional link between antimicrobial and amyloid peptides.,” Soft Matter, vol. 10, no. 38, pp. 7425–51, Oct. 2014.
27. L. Miravalle, T. Tokuda, R. Chiarle, G. Giaccone, O. Bugiani, F. Tagliavini, B. Frangione, and J. Ghiso, “Substitutions at codon 22 of Alzheimer’s abeta peptide induce diverse conformational changes and apoptotic effects in human cerebral endothelial cells.,” J. Biol. Chem., vol. 275, no. 35, pp. 27110–6, Sep. 2000.
28. Y. Fezoui and D. B. Teplow, “Kinetic studies of amyloid beta-protein fibril assembly. Differential effects of alpha-helix stabilization.,” J. Biol. Chem., vol. 277, no. 40, pp. 36948–54, Oct. 2002.
    1. E. Roher, J. D. Lowenson, S. Clarke, A. S. Woods, R. J. Cotter, E. Gowing, and M. J. Ball, “beta-Amyloid-(1-42) is a major component of cerebrovascular amyloid deposits: implications for the pathology of Alzheimer disease.,” Proc. Natl. Acad. Sci. U. S. A., vol. 90, no. 22, pp. 10836–10840, Nov. 1993.
29. S. J. Soscia, J. E. Kirby, K. J. Washicosky, S. M. Tucker, M. Ingelsson, B. Hyman, M. a Burton, L. E. Goldstein, S. Duong, R. E. Tanzi, and R. D. Moir, “The Alzheimer’s disease-associated amyloid beta-protein is an antimicrobial peptide.,” PLoS One, vol. 5, no. 3, p. e9505, Jan. 2010.
30. G. G. Tartaglia, A. Cavalli, R. Pellarin, and A. Caflisch, “Prediction of Aggregation Rate and Aggregation-Prone Segments in Polypeptide Sequences,” Fakultat der Universitat Zurich, 2005.
31. S. Jones, J. Manning, N. M. Kad, and S. E. Radford, “Amyloid-forming peptides from beta2-microglobulin-Insights into the mechanism of fibril formation in vitro.,” J. Mol. Biol., vol. 325, no. 2, pp. 249–257, Jan. 2003.
32. R. Vidal, T. Revesz, A. Rostagno, E. Kim, J. L. Holton, T. Bek, M. Bojsen-Møller, H. Braendgaard, G. Plant, J. Ghiso, and B. Frangione, “A decamer duplication in the 3’ region of the BRI gene originates an amyloid peptide that is associated with dementia in a Danish kindred.,” Proc. Natl. Acad. Sci. U. S. A., vol. 97, no. 9, pp. 4920–5, Apr. 2000.
33. R. Tycko and Y. Ishii, “Constraints on supramolecular structure in amyloid fibrils from two-dimensional solid-state NMR spectroscopy with uniform isotopic labeling.,” J. Am. Chem. Soc., vol. 125, no. 22, pp. 6606–6607, Jun. 2003.
34. B. Häggqvist, J. Näslund, K. Sletten, G. T. Westermark, G. Mucchiano, L. O. Tjernberg, C. Nordstedt, U. Engström, and P. Westermark, “Medin: an integral fragment of aortic smooth muscle cell-produced lactadherin forms the most common human amyloid.,” Proc. Natl. Acad. Sci. U. S. A., vol. 96, no. 15, pp. 8669–8674, Jul. 1999.
35. S. Farnaud and R. W. Evans, “Lactoferrin - A multifunctional protein with antimicrobial properties.,” Mol. Immunol., vol. 40, no. 7, pp. 395–405, Nov. 2003.
36. P. M. Hwang, N. Zhou, X. Shan, C. H. Arrowsmith, and H. J. Vogel, “Three-dimensional solution structure of lactoferricin B, an antimicrobial peptide derived from bovine lactoferrin.,” Biochemistry, vol. 37, no. 12, pp. 4288–98, Mar. 1998.
37. H. Zhao, A. Jutila, T. Nurminen, S. a Wickström, J. Keski-Oja, and P. K. J. Kinnunen, “Binding of endostatin to phosphatidylserine-containing membranes and formation of amyloid-like fibers.,” Biochemistry, vol. 44, no. 8, pp. 2857–63, Mar. 2005.
    1. K. Mahalka and P. K. J. Kinnunen, “Binding of amphipathic alpha-helical antimicrobial peptides to lipid membranes: lessons from temporins B and L.,” Biochim. Biophys. Acta, vol. 1788, no. 8, pp. 1600–9, Aug. 2009.
38. H. Zhao, R. Sood, A. Jutila, S. Bose, G. Fimland, J. Nissen-Meyer, and P. K. J. Kinnunen, “Interaction of the antimicrobial peptide pheromone Plantaricin A with model membranes: implications for a novel mechanism of action.,” Biochim. Biophys. Acta, vol. 1758, no. 9, pp. 1461–74, Sep. 2006.
39. O. Lequin, A. Ladram, L. Chabbert, F. Bruston, O. Convert, D. Vanhoye, G. Chassaing, P. Nicolas, and M. Amiche, “Dermaseptin S9, an alpha-helical antimicrobial peptide with a hydrophobic core and cationic termini.,” Biochemistry, vol. 45, no. 2, pp. 468–80, Jan. 2006.
40. P. C. A. van der Wel, J. R. Lewandowski, and R. G. Griffin, “Structural characterization of GNNQQNY amyloid fibrils by magic angle spinning NMR.,” Biochemistry, vol. 49, no. 44, pp. 9457–9469, Nov. 2010.
41. P. Hammarström, X. Jiang, A. R. Hurshman, E. T. Powers, and J. W. Kelly, “Sequence-dependent denaturation energetics: A major determinant in amyloid disease diversity.,” Proc. Natl. Acad. Sci. U. S. A., vol. 99 Suppl 4, pp. 16427–32, Dec. 2002.
42. F. Tagliavini, F. Prelli, J. Ghiso, O. Bugiani, D. Serban, S. B. Prusiner, M. R. Farlow, B. Ghetti, and B. Frangione, “Amyloid protein of Gerstmann-Sträussler-Scheinker disease (Indiana kindred) is an 11 kd fragment of prion protein with an N-terminal glycine at codon 58.,” EMBO J., vol. 10, no. 3, pp. 513–519, Mar. 1991.
43. L. L. Iversen, R. J. Mortishire-Smith, S. J. Pollack, and M. S. Shearman, “The toxicity in vitro of beta-amyloid protein.,” Biochem. J., vol. 311 (Pt 1), pp. 1–16, Oct. 1995.
44. E. Hughes, R. M. Burke, and A. J. Doig, “Inhibition of toxicity in the beta-amyloid peptide fragment beta -(25-35) using N-methylated derivatives: a general strategy to prevent amyloid formation.,” J. Biol. Chem., vol. 275, no. 33, pp. 25109–25115, Aug. 2000.
45. F. Chiti, N. Taddei, F. Baroni, C. Capanni, M. Stefani, G. Ramponi, and C. M. Dobson, “Kinetic partitioning of protein folding and aggregation.,” Nat. Struct. Biol., vol. 9, no. 2, pp. 137–43, Feb. 2002.
46. G. Shanmugam, N. Phambu, and P. L. Polavarapu, “Unusual structural transition of antimicrobial VP1 peptide.,” Biophys. Chem., vol. 155, no. 2–3, pp. 104–8, May 2011.
47. L. Skora, S. Becker, and M. Zweckstetter, “Characterization of amyloid fibrils of human beta-2-microglobulin by high-resolution magic-angle spinning NMR.,” Chembiochem, vol. 11, no. 13, pp. 1829–1832, Sep. 2010.
48. K. Yamamoto, H. Yagi, Y.-H. Lee, J. Kardos, Y. Hagihara, H. Naiki, and Y. Goto, “The amyloid fibrils of the constant domain of immunoglobulin light chain.,” FEBS Lett., vol. 584, no. 15, pp. 3348–3353, Aug. 2010.
49. C. Goldsbury, K. Goldie, J. Pellaud, J. Seelig, P. Frey, S. a Müller, J. Kistler, G. J. Cooper, and U. Aebi, “Amyloid fibril formation from full-length and fragments of amylin.,” J. Struct. Biol., vol. 130, no. 2–3, pp. 352–62, Jun. 2000.
50. P. Westermark, C. Wernstedt, E. Wilander, D. W. Hayden, T. D. O’Brien, and K. H. Johnson, “Amyloid fibrils in human insulinoma and islets of Langerhans of the diabetic cat are derived from a neuropeptide-like protein also present in normal islet cells.,” Proc. Natl. Acad. Sci. U. S. A., vol. 84, no. 11, pp. 3881–3885, Jun. 1987.
    1. N. Roberts, B. Leighton, J. A. Todd, D. Cockburn, P. N. Schofield, R. Sutton, S. Holt, Y. Boyd, A. J. Day, and E. A. Foot, “Molecular and functional characterization of amylin, a peptide associated with type 2 diabetes mellitus.,” Proc. Natl. Acad. Sci. U. S. A., vol. 86, no. 24, pp. 9662–9666, Dec. 1989.
    2. Wasmer, A. Lange, H. Van Melckebeke, A. B. Siemer, R. Riek, and B. H. Meier, “Amyloid fibrils of the HET-s(218-289) prion form a beta solenoid with a triangular hydrophobic core.,” Science, vol. 319, no. 5869, pp. 1523–6, Mar. 2008.
51. H. Van Melckebeke, C. Wasmer, A. Lange, E. A. B., A. Loquet, A. Böckmann, and B. H. Meier, “Atomic-resolution three-dimensional structure of HET-s(218-289) amyloid fibrils by solid-state NMR spectroscopy.,” J. Am. Chem. Soc., vol. 132, no. 39, pp. 13765–75, Oct. 2010.
52. J. J. Balbach, Y. Ishii, O. N. Antzutkin, R. D. Leapman, N. W. Rizzo, F. Dyda, J. Reed, and R. Tycko, “Amyloid fibril formation by A beta 16-22, a seven-residue fragment of the Alzheimer’s beta-amyloid peptide, and structural characterization by solid state NMR.,” Biochemistry, vol. 39, no. 45, pp. 13748–13759, Nov. 2000.
53. M. Salmona, P. Malesani, L. De Gioia, S. Gorla, M. Bruschi, A. Molinari, F. Della Vedova, B. Pedrotti, M. A. Marrari, T. Awan, O. Bugiani, G. Forloni, and F. Tagliavini, “Molecular determinants of the physicochemical properties of a critical prion protein region comprising residues 106-126.,” Biochem. J., vol. 342 ( Pt 1, pp. 207–14, Aug. 1999.
54. D. R. Booth, M. Sunde, V. Bellotti, C. V Robinson, W. L. Hutchinson, P. E. Fraser, P. N. Hawkins, C. M. Dobson, S. E. Radford, C. C. Blake, and M. B. Pepys, “Instability, unfolding and aggregation of human lysozyme variants underlying amyloid fibrillogenesis.,” Nature, vol. 385, no. 6619, pp. 787–93, Feb. 1997.
55. R. Sood, Y. A. Domanov, M. Pietiäinen, V. P. Kontinen, and P. K. J. Kinnunen, “Binding of LL-37 to model biomembranes: insight into target vs host cell recognition.,” Biochim. Biophys. Acta, vol. 1778, no. 4, pp. 983–96, Apr. 2008.
56. R. Gössler-Schöfberger, G. Hesser, M. Muik, C. Wechselberger, and A. Jilek, “An orphan dermaseptin from frog skin reversibly assembles to amyloid-like aggregates in a pH-dependent fashion.,” FEBS J., vol. 276, no. 20, pp. 5849–59, Oct. 2009.
57. E. H. Koo, P. T. Lansbury, and J. W. Kelly, “Amyloid diseases: abnormal protein aggregation in neurodegeneration.,” Proc. Natl. Acad. Sci. U. S. A., vol. 96, no. 18, pp. 9989–90, Aug. 1999.
58. R. Azriel and E. Gazit, “Analysis of the minimal amyloid-forming fragment of the islet amyloid polypeptide. An experimental support for the key role of the phenylalanine residue in amyloid formation.,” J. Biol. Chem., vol. 276, no. 36, pp. 34156–34161, Sep. 2001.
    1. Larsson, L. Söderberg, G. T. Westermark, K. Sletten, U. Engström, L. O. Tjernberg, J. Näslund, and P. Westermark, “Unwinding fibril formation of medin, the peptide of the most common form of human amyloid.,” Biochem. Biophys. Res. Commun., vol. 361, no. 4, pp. 822–828, Oct. 2007.
59. M. Torrent, F. Odorizzi, M. V. Nogués, and E. Boix, “Eosinophil cationic protein aggregation: identification of an N-terminus amyloid prone region.,” Biomacromolecules, vol. 11, no. 8, pp. 1983–90, Aug. 2010.
60. Y. Ohhashi, K. Hasegawa, H. Naiki, and Y. Goto, “Optimum amyloid fibril formation of a peptide fragment suggests the amyloidogenic preference of beta2-microglobulin under physiological conditions.,” J. Biol. Chem., vol. 279, no. 11, pp. 10814–10821, Mar. 2004.
61. P. Westermark, U. Engström, K. H. Johnson, G. T. Westermark, and C. Betsholtz, “Islet amyloid polypeptide: pinpointing amino acid residues linked to amyloid fibril formation.,” Proc. Natl. Acad. Sci. U. S. A., vol. 87, no. 13, pp. 5036–5040, Jul. 1990.
62. L. Goldschmidt, P. K. Teng, R. Riek, and D. Eisenberg, “Identifying the amylome, proteins capable of forming amyloid-like fibrils.,” Proc. Natl. Acad. Sci. U. S. A., vol. 107, no. 8, pp. 3487–3892, Feb. 2010.
63. M. R. Nilsson and D. P. Raleigh, “Analysis of amylin cleavage products provides new insights into the amyloidogenic region of human amylin.,” J. Mol. Biol., vol. 294, no. 5, pp. 1375–1385, Dec. 1999.
64. C. Exley, E. House, T. Patel, L. Wu, and P. E. Fraser, “Human pro-islet amyloid polypeptide (ProIAPP(1-48)) forms amyloid fibrils and amyloid spherulites in vitro.,” J. Inorg. Biochem., vol. 104, no. 10, pp. 1125–1129, Oct. 2010.
65. C. L. Teoh, H. Yagi, M. D. W. Griffin, Y. Goto, and G. J. Howlett, “Visualization of polymorphism in apolipoprotein C-II amyloid fibrils.,” J. Biochem., vol. 149, no. 1, pp. 67–74, Jan. 2011.
66. Mazzaglia, N. Micali, L. M. Scolaro, F. Attanasio, A. Magrí, G. Pappalardo, and V. Villari, “Aggregation properties of the peptide fragments derived from the 17-29 region of the human and rat IAPP: a comparative study with two PEG-conjugated variants of the human sequence.,” J. Phys. Chem. B, vol. 114, no. 2, pp. 705–713, Jan. 2010.
67. E. MacPhee and C. M. Dobson, “Formation of Mixed Fibrils Demonstrates the Generic Nature and Potential Utility of Amyloid Nanostructures,” J. Am. Chem. Soc., vol. 122, no. 51, pp. 12707–12713, Dec. 2000.
