# Supplementary material for: Prediction of Peptide and Protein Propensity for Amyloid Formation
Source: PLoS One. 2015 Aug 4;10(8):e0134679. doi: 10.1371/journal.pone.0134679 (PMC4524629; doi:10.1371/journal.pone.0134679)
Supplement: S1 Table — (DOCX) [file pone.0134679.s003.docx]

## S3 Table. Orthogonal encoding of the amino acids

| A  R  N  D  C  Q  E  G  H  I  L  K  M  F  P  S  T  W  Y  V | =  =  =  =  =  =  =  =  =  =  =  =  =  =  =  =  =  =  =  = | [ 1 0 0 0 0 0 0 0 0 0 0 0 0 0 0 0 0 0 0 0 ]  [ 0 1 0 0 0 0 0 0 0 0 0 0 0 0 0 0 0 0 0 0 ]  [ 0 0 1 0 0 0 0 0 0 0 0 0 0 0 0 0 0 0 0 0 ]  [ 0 0 0 1 0 0 0 0 0 0 0 0 0 0 0 0 0 0 0 0 ]  [ 0 0 0 0 1 0 0 0 0 0 0 0 0 0 0 0 0 0 0 0 ]  [ 0 0 0 0 0 1 0 0 0 0 0 0 0 0 0 0 0 0 0 0 ]  [ 0 0 0 0 0 0 1 0 0 0 0 0 0 0 0 0 0 0 0 0 ]  [ 0 0 0 0 0 0 0 1 0 0 0 0 0 0 0 0 0 0 0 0 ]  [ 0 0 0 0 0 0 0 0 1 0 0 0 0 0 0 0 0 0 0 0 ]  [ 0 0 0 0 0 0 0 0 0 1 0 0 0 0 0 0 0 0 0 0 ]  [ 0 0 0 0 0 0 0 0 0 0 1 0 0 0 0 0 0 0 0 0 ]  [ 0 0 0 0 0 0 0 0 0 0 0 1 0 0 0 0 0 0 0 0 ]  [ 0 0 0 0 0 0 0 0 0 0 0 0 1 0 0 0 0 0 0 0 ]  [ 0 0 0 0 0 0 0 0 0 0 0 0 0 1 0 0 0 0 0 0 ]  [ 0 0 0 0 0 0 0 0 0 0 0 0 0 0 1 0 0 0 0 0 ]  [ 0 0 0 0 0 0 0 0 0 0 0 0 0 0 0 1 0 0 0 0 ]  [ 0 0 0 0 0 0 0 0 0 0 0 0 0 0 0 0 1 0 0 0 ]  [ 0 0 0 0 0 0 0 0 0 0 0 0 0 0 0 0 0 1 0 0 ]  [ 0 0 0 0 0 0 0 0 0 0 0 0 0 0 0 0 0 0 1 0 ]  [ 0 0 0 0 0 0 0 0 0 0 0 0 0 0 0 0 0 0 0 1 ] |
| --- | --- | --- |
