# Supplementary material for: Prediction of Peptide and Protein Propensity for Amyloid Formation
Source: PLoS One. 2015 Aug 4;10(8):e0134679. doi: 10.1371/journal.pone.0134679 (PMC4524629; doi:10.1371/journal.pone.0134679)
Supplement: S2 Table — (DOCX) [file pone.0134679.s004.docx]

# S4 Table. Input parameters and consideration made to classify a sequence as amyloidogenic from the provided outputs, organized by prediction method.

| Method | Input parameters | Considerations made |
| --- | --- | --- |
| Aggrescan | None required. | A sequence is considered amyloidogenic if there is at least one predicted hotspot. |
| Amylpred | None required | A sequence is considered amyloidogenic if there is at least one predicted amyloidogenic region. |
| Amylpred2 | Methods to be used: 'AGGRESCAN', 'NETCSSP', 'AMYLMUTS', 'PAFIG', 'AMYLPATTERN', 'SECSTR', 'APD', 'TANGO', 'BSC', 'WALTZ', 'CONFENERGY' | A sequence is considered amyloidogenic if there is at least one predicted amyloidogenic region (Hit). |
| Foldamyloid | Scale: expected number of contacts 8A  Averaging frame: 5  Reliable frame: 5  Threshold: 21.4 | A sequence is considered amyloidogenic if there is at least one region predicted as amyloidogenic. According with the authors, “a region is predicted as being amyloidogenic if the average value of the parameter over this region is greater than threshold and the region is greater or equal in size to the reliable frame”. |
| MetAmyl | Threshold: Best global accuracy | A sequence is considered amyloidogenic if there is at least one predicted hotspot. |
| Pafig | Minimum hotspot length: 5 | A sequence is considered amyloidogenic if there is at least one predicted hotspot. Note: once all hotspot information files were blank, a sequence was considered amyloidogenic if any of the predicted windows had a value equal or higher than the threshold showed in the output plots (0.5. |
| Pasta | Npair: 100 | A sequence is considered amyloidogenic if the pasta energy for the lowest predicted pairing is lower or equal to the threshold stated by the authors (-4.0). |
| Pasta 2 | Top x energy pairings: 10,  Mode: Self pairing | A sequence is considered amyloidogenic if the pasta energy for the lowest predicted pairing is lower or equal to the threshold stated by the authors (-4.0). |
| Tango | Protection at the N-terminus): N (No)  Protection at the C-terminus): N (No)  pH: 7  Temperature: 298.15 K  Ionic strength: 0.02 | A sequence is considered amyloidogenic if at least one hotspot is identified within the sequence, according to the authors, a hotspot is any segment has an aggregation tendency above 5.0% over 5 residues. |
| Waltz | Threshold: 0  pH: 7.0  Output: text_long_graph | A sequence is considered amyloidogenic if at least one region is predicted amyloidogenic, according to the authors, if there is at least one classification windows with a value equal or higher than the threshold for the Best Overall Performance (92). |
| Zyggregator | pH: 7.0  Propensity: zagg | A sequence is considered amyloidogenic if there is at least one predicted amyloidogenic region, according with the authors an amyloidogenic region is defined by the minimum of 5 consecutive residues with an aggregation value higher than the defined threshold of 1. |
